# Rab GTPases drive ligand‐independent NOTCH1 activation via altered endocytic trafficking in chronic lymphocytic leukemia

**DOI:** 10.1002/hem3.70440

**Published:** 2026-07-28

**Authors:** Filomena De Falco, Beatrice Del Papa, Daniele Sorcini, Letizia Valmarini, Francesco Maria Adamo, Andrea Atzeni, Angela Esposito, Estevão Carlos Silva Barcelos, Erica Dorillo, Arianna Stella, Roberta Arcaleni, Fabio Gurrieri, Miriam Pugliese, Gabriele Astolfi, Maria Paola Martelli, Mauro Di Ianni, Emanuela Rosati, Paolo Sportoletti

**Affiliations:** ^1^ Department of Medicine and Surgery Institute of Hematology and Center for Hemato‐Oncology Research (CREO), University of Perugia and Santa Maria della Misericordia Hospital Perugia Italy; ^2^ Department of Medicine and Sciences of Aging “G. d'Annunzio” University of Chieti‐Pescara Chieti Italy; ^3^ Department of Oncology and Hematology Ospedale Civile “Santo Spirito”, ASL Pescara Pescara Italy; ^4^ Department of Medicine and Surgery University of Perugia Perugia Italy

## Abstract

Clinically relevant NOTCH1 activation is frequently observed in chronic lymphocytic leukemia (CLL), even in the absence of gene mutations, raising questions about the underlying mechanisms. In CLL cells, NOTCH1 activation may occur through mutation‐independent mechanisms involving ligand interaction or cell‐intrinsic, ligand‐independent pathways that are not yet fully elucidated. To explore ligand‐independent activation, we examined the involvement of NOTCH1 endocytic trafficking in generating the active intracellular domain (N1‐ICD) in CLL cells. Using proximity ligation assay, we demonstrated that the NOTCH1 extracellular domain (N1‐ECD), transmembrane subunit (N1‐TM), and N1‐ICD colocalize with Rab5 and Rab7, indicating NOTCH1 internalization and cleavage within endosomal compartments. Experiments with the endocytosis inhibitor Pitstop‐2 demonstrated that NOTCH1 internalization is essential for N1‐ICD generation. We provided evidence that N1‐ICD generation occurs in Rab5 and Rab7 endosomal membranes. Treatment with chloroquine reduced N1‐ICD, due to impaired endosomal acidification affecting enzymatic activity. Presenilin‐1, the catalytic subunit of the γ‐secretase complex responsible for N1‐ICD generation, was found in early endosome compartments and colocalized with Rab5, Rab7, N1‐TM, and N1‐ICD. CLL cells expressing N1‐ICD showed higher Rab5, Rab7, and presenilin‐1 levels, increased Rab5 membrane association, and presenilin‐1 activity versus N1‐ICD‐negative cells which showed increased lysosomal targeting. Silencing Rab5 or Rab7 by siRNA, or inhibiting Rab prenylation with psoromic acid, led to reduced N1‐ICD levels and increased apoptosis in CLL cells. Interestingly, NOTCH1‐mutated cases showed similar NOTCH1 trafficking. These findings identify for the first time a Rab‐dependent endocytic trafficking as a key regulator of NOTCH1 activation and a potential therapeutic target in CLL.

## Graphical abstract

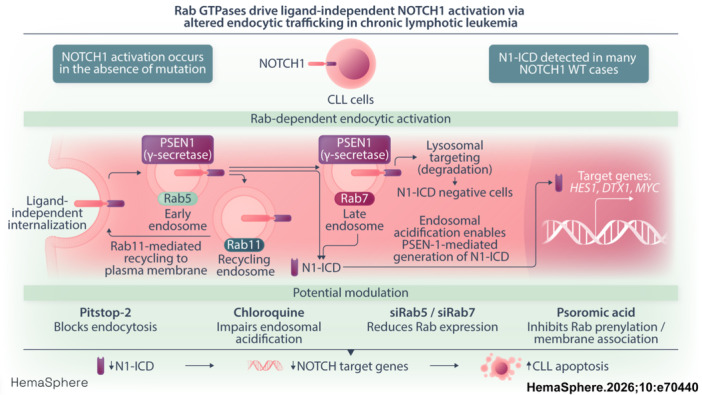

## INTRODUCTION

Deregulated NOTCH1 signaling is associated with several human cancers due to genetic abnormalities, alterations in the regulatory pathways, and aberrant expression of NOTCH ligands within the tumor microenvironment.^1^, ^2^, ^3^ Increasing evidence demonstrated that *NOTCH1* is a key cancer gene involved in the pathogenesis of chronic lymphocytic leukemia (CLL),^4^ which still remains incurable despite available therapies.^5^, ^6^, ^7^, ^8^ Mutations in *NOTCH1* are the most common genetic lesion in CLL patients with poor outcome, where the mutation potentiates NOTCH1 signaling.^9^, ^10^, ^11^, ^12^, ^13^, ^14^, ^15^ Recent evidence revealed that NOTCH1 can be also found activated independently of the mutational status^16^, ^17^ in CLL. Indeed, in the vast majority of patients, leukemic cells have a constitutive NOTCH1/2 activation, which sustains their apoptosis resistance.^18^, ^19^, ^20^ Previous studies have shown that NOTCH1/JAGGED1 interactions among circulating CLL cells lacking *NOTCH1* mutations are not responsible for NOTCH1 activation,^21^ suggesting that this activation may result from cell‐intrinsic mechanisms independent of ligand binding.^22^ Activation of the Notch pathway occurs through three sequential proteolytic cleavages (S1–S3). NOTCH receptors are synthesized in the endoplasmic reticulum (ER) and trafficked to the Golgi apparatus, where S1 cleavage by furin generates a heterodimeric receptor that is transported to the plasma membrane. Upon ligand binding, an ADAM metalloprotease mediates S2 cleavage, generating a membrane‐tethered intermediate that is subsequently processed by the γ‐secretase complex (S3), leading to the release of the active NOTCH intracellular domain (ICD), which translocates to the nucleus to activate target gene transcription.

In physiologic conditions, when ligand interactions are absent, NOTCH1 receptor is internalized in the endocytic compartments and can be either recycled to the plasma membrane via recycling endosomes or sorted to the late endosomes and lysosomes for activation or degradation.^23^, ^24^ A well‐regulated endosomal sorting exerts a crucial role in preventing improper NOTCH1 activation, but if some regulators or components of intracellular trafficking are altered, NOTCH1 can be aberrantly activated resulting in disease.^25^, ^26^

Rab proteins are master regulators of intracellular membrane trafficking and dynamic compartmentalization of the endomembrane system^27^, ^28^ therefore representing potential critical factors involved in sorting NOTCH1 into activation or degradative pathways. Rab5 is a rate‐limiting catalyst of the endocytic uptake and delivery to the early endosomes (EEs), while Rab7 controls transport from EEs to late endosomes and from late endosomes to lysosomes, where NOTCH1 can be either activated at the vesicle membranes or degraded within their lumen. Conversely, Rab11 controls recycling to the plasma membrane.^27^, ^28^ In order to localize to inner membranes and perform their functions, Rab proteins need to be prenylated by Rab geranylgeranyl transferase (GGTase II), an enzyme of the mevalonate pathway which catalyzes the addition of two geranylgeranyl groups at the C‐terminal (CT) cysteine residues of Rab proteins.^29^, ^30^, ^31^, ^32^ Increasing evidence indicates that alterations in Rab expression, activity, and regulation are associated with cancer progression and poor prognosis in several malignancies.^33^, ^34^, ^35^, ^36^, ^37^

Small molecule inhibitors and antibodies targeting NOTCH signaling have been developed and explored in clinical trials for cancer treatment, but they failed due to limited anti‐tumor efficacy and toxicity in normal tissues.^38^ Therefore, the discovery of novel regulators of malignant NOTCH signaling represents a challenge for identifying novel NOTCH‐based therapeutics in CLL^39^ and other NOTCH‐related cancers.^40^

In this study, we investigated new ligand‐independent mechanisms of NOTCH1 activation involving Rab5 and Rab7 in CLL cells. We showed that NOTCH1 is internalized into endocytic vesicles of CLL cells and cleaved into the active NOTCH1‐intracellular domain (N1‐ICD) form on vesicular membranes. Inappropriate activation of NOTCH1 signaling was associated with alterations of Rab5, Rab7, and presenilin‐1 (PSEN1), the active subunit of the γ‐secretase complex responsible for NOTCH1 cleavage. Pharmacological interference with Rab geranylgeranylation impaired the generation of active N1‐ICD and reduced CLL cell viability. These findings reveal a previously unknown mechanism of NOTCH1 activation in CLL providing new potential targets for therapy.

## MATERIAL AND METHODS

### CLL patients and clinical laboratory characteristics

Forty‐two CLL patients were included in the analysis. Diagnoses of CLL were based on Stanford criteria defined by the National Cancer Institute‐sponsored Working Group,^41^ and clinical staging was based on the Rai and Binet classifications.^42^ This study was approved by the local Ethics Committee, and all patients signed informed consent in accordance with the Declaration of Helsinki. CD19^+^/CD5^+^ CLL cells were isolated from peripheral blood as reported,^18^, ^19^, ^20^ viably frozen and thawed immediately prior to each experiment. All CLL samples contained more than 70% CD19^+^/CD5^+^ CLL cells, assessed by flow cytometry (EPICS‐XL‐MCL; Beckman Coulter)*. IGHV* mutations, cytogenetic abnormalities, *TP53*, and *NOTCH1* exon 34 mutational status were analyzed as reported.^13^, ^18^, ^43^ *NOTCH1 3′UTR*, *SF3B1*, *FBXW7*, and *MED12* mutations were examined by NGS (Supplemental Information). Table S1 gives the clinical and biological characteristics of CLL patients.

### CLL cell treatments

Primary CLL cells (2 × 10^6^/mL), resuspended in complete medium consisting of RPMI 1640 supplemented with 10% heat‐inactivated fetal bovine serum (Gibco), 2mM l‐glutamine, 100 U/mL penicillin, and 100 μg/mL streptomycin (all from Invitrogen), were cultured for indicated times with the endocytosis inhibitor pitstop‐2 (Abcam) and the GGTase II inhibitor psoromic acid (PA; Santa Cruz) or dimethyl sulfoxide (DMSO) as control, and with chloroquine (CHQ; Sigma) or complete medium as control.

### Small interfering RNA transfection

To downregulate Rab5 or Rab7 expression, CLL cells were transfected using the Amaxa nucleofection technology and the ON‐TARGETplus SMARTpool small interfering RNA (siRNA) to Rab5 (siRab5), Rab7 (siRab7), or ON‐TARGETplus siCONTROL nontargeting pool (siCtrl) as a negative control (Dharmacon RNA Technologies). CLL cells (12 × 10^6^) were resuspended in 100 µL Cell Line Solution Kit V (Lonza Group Ltd) with 0.25 μM of the specific siRab or siCtrl, transferred to the cuvettes, and transfected with the Amaxa Nucleofector II/2b device (program U‐013). Cells were transferred into 12‐well plates in complete medium, and after 48 h were examined for the expression of N1‐TM, N1‐ICD, PSEN1, and the specific Rab protein.

### Protein extract preparation and Western blot analysis

Whole‐cell lysates were extracted from CLL cells as reported previously.^18^, ^19^, ^20^ Cytoplasmic and membrane subcellular fractions were isolated using a Mem‐PER^TM^ plus kit (Thermo Fisher Scientific). EEs were isolated using Trident Endosome Isolation Kit (GeneTex), according to the manufacturer's instructions. Western blot analysis was performed using the primary antibodies listed in Table S2. Primary antibodies were detected using horseradish peroxidase‐conjugated secondary antibodies (Jackson ImmunoResearch Laboratories) together with the LiteAblot®TURBO Enhanced Chemiluminescent Substrate (EuroClone Life Sciences) or the SuperSignal™ West Pico PLUS Chemiluminescent Substrate (Thermo Fisher Scientific). Densitometric analysis was performed using Quantity One or Image Lab software (Bio‐Rad).

### Quantitative real‐time polymerase chain reaction

RNA was extracted using RNeasy Plus Kit (Qiagen), and cDNA was obtained using Prime Script RT Master Mix (Takara Bio). Real‐time quantitative polymerase chain reaction (PCR) was performed with PCR Master Mix Power SYBR Green (Applied Biosystems), using the 7900HT Fast Real‐Time PCR System (Applied Biosystems). The sequences of primers for *RAB5*, *RAB7*, *PRESENILIN1*, *HES1*, *DTX1*, *c‐MYC*, and *GAPDH* (all from Thermo Fisher Scientific) analysis are shown in Table S3. The expression of each target gene was normalized to GAPDH, and relative fold change was calculated using the 2−ΔΔCt method.

### Analysis of cell viability/apoptosis

Cell viability was evaluated by CellTiter‐Glo® assay as reported.^44^ Cell apoptosis was assessed by flow cytometry after Annexin V‐APC/propidium iodide (An V/PI) double staining, performed with a commercial kit (Immunotech), according to the manufacturer's instructions. Results were analyzed by FlowJo software.

### Proximity ligation assay and confocal microscopy analysis

The proximity ligation assay (PLA) was applied to examine the interactions of (i) N1‐ECD, N1‐TM, and N1‐ICD with Rab5, Rab7, and Rab11; (ii) PSEN1 with Rab5, Rab7, N1‐TM, and N1‐ICD; and (iii) N1‐ECD and N1‐TM with LAMP1. PLA was performed on fixed CLL cells with Duolink® PLA technology probes and reagents (Sigma‐Aldrich), following the manufacturer's protocol. Briefly, CLL cells were permeabilized in 0.1% Triton X‐100 in phosphate‐buffered saline (PBS), blocked in Duolink® Blocking Solution, and incubated overnight at 4°C with the antibodies listed in Table S2, diluted in Duolink® Antibody Diluent solution. Incubation with Duolink® MINUS and PLUS probes conjugated to secondary antibody, ligation, and amplification steps for PLA were performed as suggested by the manufacturer using 40 μL volume reaction. Following amplification, slides were washed for 10 min in Buffers A and B, and then mounted with Duolink® in situ mounting medium containing DAPI. Negative controls were obtained by omitting both primary antibodies or by replacing one of the two primary antibodies in the interaction with an isotype control antibody; no PLA‐positive signals were observed (Figure S1). Positive controls were obtained by analyzing Bim/Bcl‐2 interaction (Figure S1) or PSEN1/NOTCH1 interaction. Fluorescent images were obtained using a confocal microscope LSM 800 (Zeiss) with Airyscan using a 63× oil immersion and 1.4 NA objective. Scale bar, 10 μm. For PLA quantification, positive interactions were evaluated by counting PLA‐positive cells in five independent microscopic fields per sample. The percentage of PLA‐positive cells was calculated for each field and then averaged across the five fields to obtain a single value per sample. A minimum of 20 cells per condition were analyzed, depending on cell density and sample availability.

### Statistical analyses

All data were analyzed using GraphPad v8 and v10 (GraphPad Software Inc.). Nonparametric Wilcoxon test for paired data and Mann–Whitney for unpaired data were used. In confocal microscopy analyses, paired *t*‐test or Welch's *t*‐test for unpaired data were used, as appropriate. For siRNA silencing experiments, statistical comparisons were performed using a paired *t*‐test. Results were considered statistically significant with P value < 0.05.

Additional methods are presented in the Supplemental Information.

## RESULTS

### NOTCH1 expression and activation in CLL is observed in the absence of mutations

This study included CLL cells from 40 NOTCH1 wild‐type and two NOTCH1‐mutated patients that were analyzed for NOTCH1 expression and activation. Western blot analysis of the NOTCH1‐transmembrane subunit (N1‐TM) and the active intracellular domain (N1‐ICD) showed that 28 wild‐type samples were N1‐ICD positive, while 10 expressed only N1‐TM (N1‐ICD negative), indicating receptor expression without activation (Figure S2 and Table S4). Two additional wild‐type samples lacked both N1‐TM and N1‐ICD. The two NOTCH1‐mutated samples were positive for N1‐ICD. Classification of N1‐ICD as positive or negative resulted reproducible across multiple Western blot runs. Surface expression of NOTCH1 was confirmed by flow cytometry, showing that N1‐ICD‐negative samples displayed the receptor on the plasma membrane (Table S4). To exclude potential confounding effects from alternative genetic activators of the NOTCH pathway, 31 out of 42 cases were screened for mutations in *NOTCH1 3′UTR*, *SF3B1*, *FBXW7*, and *MED12* by NGS analysis (Supplemental Methods), revealing no enrichment of these alterations in NOTCH1‐activated samples (Table S1). These data demonstrate that NOTCH1 activation can occur independently of mutations in our patient cohort.

### Colocalization of NOTCH1 subunits with Rab proteins differs between N1‐ICD‐positive and N1‐ICD‐negative CLL cells

**Figure 1:**
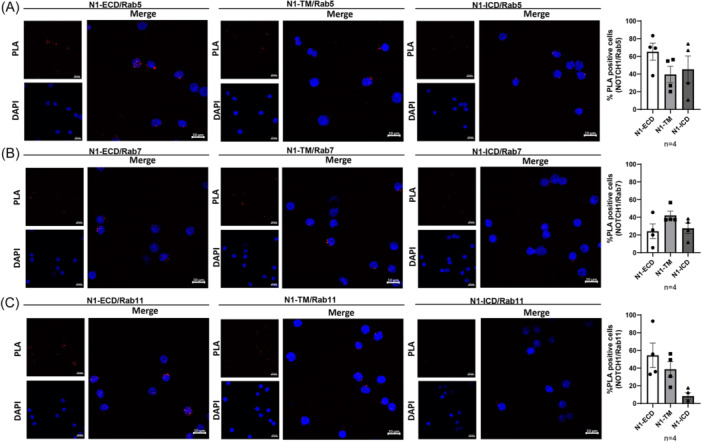
**Analysis of NOTCH1 colocalization with Rab5, Rab7, and Rab11 markers in N1‐ICD‐positive cells.** Proximity ligation assay (PLA) was performed in N1‐ICD‐positive chronic lymphocytic leukemia (CLL) cells (*n* = 4) to detect the interaction of N1‐ECD, N1‐TM, and N1‐ICD with Rab5 **(A)**, Rab7 **(B)**, and Rab11 **(C)**. Left panels, confocal microscopy images from one representative CLL sample. PLA signals indicating interactions are visualized by red spots and nuclei by DAPI staining. Images were acquired using confocal microscopy with a 63× oil immersion and 1.4 NA objective. Scale bar 10 µM. Right panels, bar graphs with individual data points showing mean ± SEM of the percentage of PLA‐positive cells in four samples. For each patient, five fields per condition were analyzed and averaged to obtain a single value.

To define the fate of NOTCH1 within the endocytic pathway, we examined the colocalization of Rab5, Rab7, and Rab11, which identify early, late, and recycling endosomes, respectively, with the three distinct subunits of the NOTCH1 receptor, including the extracellular domain (N1‐ECD), N1‐TM, and N1‐ICD, using in situ PLA. The analysis showed that N1‐ECD, N1‐TM and N1‐ICD resulted in proximity with Rab5 (with 65.3% ± 9.59%, 39.5% ± 9.3%, and 45.4% ± 15.0% positive cells, respectively) and Rab7 (24.2% ± 8.3%, 42.1% ± 4.8%, and 27.5% ± 5.9% positive cells, respectively) in N1‐ICD‐positive CLL cells (Figures 1A,B and S3) suggesting internalization of full‐length NOTCH1 protein into endocytic compartments. Conversely, Rab11 colocalized with N1‐ECD, N1‐TM, and only to a lesser extent with N1‐ICD (54.47% ± 13.8%, 38.7% ± 8.54%, and 8.3% ± 4.06% positive cells, respectively) (Figures 1C and S3), consistent with NOTCH1 receptor sorting to plasma membrane favoring new ligand interaction for potential activation.

**Figure 2:**
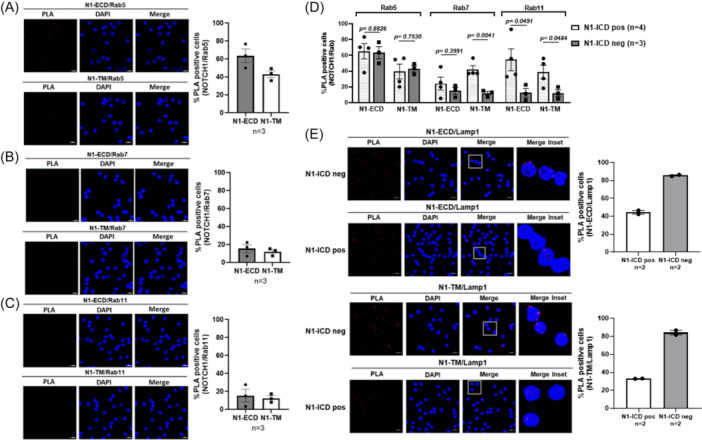
**Analysis of NOTCH1 colocalization with Rab5, Rab7, Rab11, LAMP1 markers in N1‐ICD‐negative compared to N1‐ICD‐positive cells.** Proximity ligation assay (PLA) was performed in N1‐ICD‐negative chronic lymphocytic leukemia (CLL) cells (*n* = 3) to detect the interaction of N1‐ECD, N1‐TM with Rab5 **(A)**, Rab7 **(B)**, and Rab11 **(C)**. Left panels, confocal microscopy images from one representative CLL sample. PLA signals indicating interactions are visualized by red spots and nuclei by DAPI staining. Images were acquired using confocal microscopy with a 63× oil immersion and 1.4 NA objective. Scale bar 10 µm. Right panels, bar graphs with individual data points showing mean ± SEM of the percentage of PLA‐positive cells in three samples. For each patient, five fields per condition were analyzed and averaged to obtain a single value. **(D)** Comparison between N1‐ICD‐positive (*n* = 4) and N1‐ICD‐negative (*n* = 3) CLL samples of the quantitative analysis of the PLA signals of N1‐ECD and N1‐TM colocalization with Rab5, Rab7, and Rab11 markers. Bar graphs with individual data points showing mean ± SEM. P values according to Welch's *t*‐test are indicated above each comparison. **(E)** PLA was performed in N1‐ICD‐negative CLL cells (*n* = 2) and N1‐ICD‐positive CLL cells (*n* = 2) to detect the interaction of N1‐ECD (upper panel) and N1‐TM (bottom panel) with LAMP1. Left, confocal microscopy images from one representative CLL sample per group. PLA signals indicating interactions are visualized by red spots and nuclei by DAPI staining. Images were acquired using confocal microscopy with a 63× oil immersion and 1.4 NA objective. Scale bar 10 µm. Right, bar graphs with individual data points showing mean ± SEM of the percentage of PLA‐positive cells in two samples. For each patient, five fields per condition were analyzed and averaged to obtain a single value.

In N1‐ICD‐negative CLL cells, we observed proximity of N1‐ECD and N1‐TM with Rab5 (63.4% ± 7.62% and 43% ± 3.3% positive cells, respectively), Rab7 (15.3% ± 4.4% and 11.9% ± 2.3% positive cells, respectively), and Rab11 (12.51% ± 5.7% and 12.3% ± 4.3% positive cells, respectively) (Figures 2A–C and S4). Comparison of PLA data from N1‐ICD‐positive and ‐negative CLL samples showed no significant differences in the percentage of N1‐ECD/Rab5 and N1‐TM/Rab5 PLA‐positive cells, suggesting that the NOTCH1 receptor is endocytosed independently of its activation (Figure 2D). In contrast, the percentage of N1‐ECD/Rab7‐ and N1‐TM/Rab7‐positive cells was higher in N1‐ICD‐positive samples compared to N1‐ICD‐negative ones (Figure 2D), consistent with a different endocytic fate of NOTCH1. The percentages of N1‐ECD/Rab11 and N1‐TM/Rab11‐positive cells were significantly lower in N1‐ICD‐negative than in N1‐ICD‐positive cells, suggesting reduced NOTCH1 recycling to the plasma membrane (Figure 2D). To further investigate the intracellular fate of NOTCH1 in ICD‐negative CLL cells, we assessed the association of N1‐ECD and N1‐TM with the lysosomal marker LAMP1 by PLA in both N1‐ICD‐negative and N1‐ICD‐positive samples. Quantitative analysis revealed a higher percentage of PLA‐positive cells for both N1‐ECD/LAMP1 and N1‐TM/LAMP1 in N1‐ICD‐negative cells compared with N1‐ICD‐positive cells (Figure 2E). These findings indicate increased proximity of NOTCH1 to lysosomal compartments in ICD‐negative cells, consistent with enhanced lysosomal targeting of the receptor.

### Association of NOTCH1 subunits with Rab proteins differs in the presence of NOTCH ligand

To compare NOTCH1 colocalization with Rab proteins under ligand‐dependent and ligand‐independent conditions, we performed PLA analyses in primary N1‐ICD‐positive CLL cells cultured in the presence or absence of OP9‐DLL1 cells. We observed reduced interactions of all NOTCH1 subunits with Rab5, Rab7, and Rab11 in co‐cultured cells compared to CLL cells cultured alone and increased N1‐ICD levels (Figure S5). Notably, the interactions of both N1‐TM and N1‐ICD with PSEN1, the catalytic γ‐secretase subunit, used as a positive control, were preserved under both conditions (Figure S6). Overall, these findings suggest that ligand stimulation alters the association of NOTCH1 with Rab‐positive compartments possibly by promoting rapid γ‐secretase‐mediated receptor processing and reducing its residence time within these compartments.

### NOTCH1 activation decreases upon inhibition of early endocytosis in CLL cells

**Figure 3:**
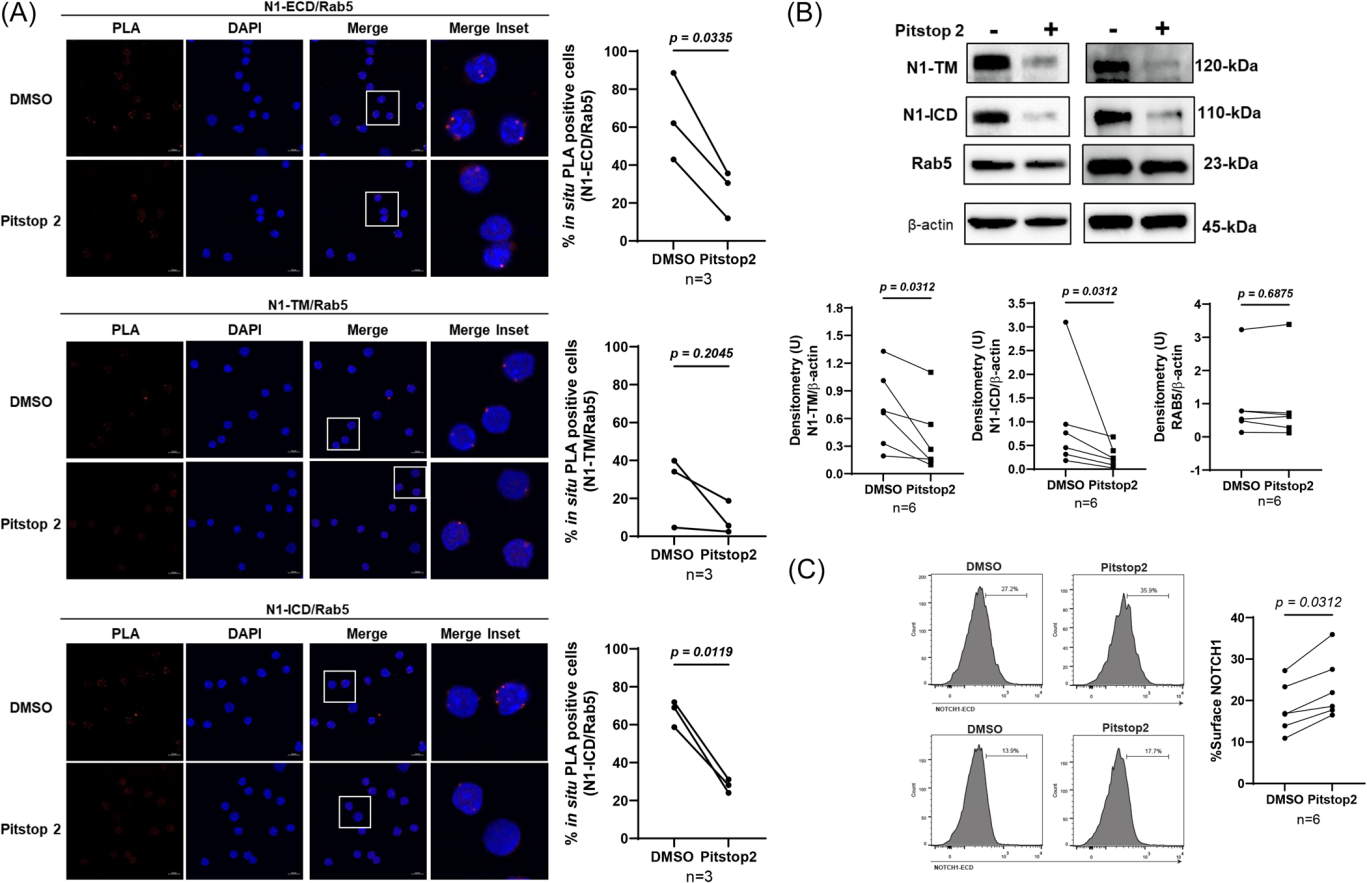
**Targeting endocytosis reduces N1‐ICD levels in chronic lymphocytic leukemia (CLL) cells**. **(A–C)** CLL cells were treated with Pitstop‐2 or dimethyl sulfoxide (DMSO) as a control for 30 min. **(A)** Proximity ligation assay (PLA) performed to detect the interactions of N1‐ECD, N1‐TM, and N1‐ICD with the early endosome marker Rab5. Left panels, confocal microscopy images from one representative CLL sample. PLA signals indicating interactions are visualized by red spots and nuclei by DAPI staining. Images were acquired using confocal microscopy with a 63× oil immersion objective (NA 1.4). Scale bar 10 µm. Right panels, dot and line diagrams showing the percentage of PLA‐positive cells from three independent CLL samples. Each dot represents one patient sample and lines connect paired conditions (DMSO vs. Pitstop‐2). For each patient, five fields per condition were analyzed and averaged to obtain a single value prior to statistical analysis. P values were calculated using a paired *t*‐test. **(B)** Western blot analysis of N1‐TM, N1‐ICD, and Rab5 expression. Upper panel, two representative CLL samples are shown. Bottom panels, dot and line diagrams showing densitometry analysis of N1‐TM, N1‐ICD, and Rab5 normalized to β‐actin (*n* = 6). P values are indicated above each graph according to the Wilcoxon paired test. **(C)** Flow cytometry analysis of surface NOTCH1 levels. Left panel, flow cytometry histograms of two representative CLL samples are shown. Right, dot and line diagrams showing the percentage of the surface NOTCH1 (*n* = 6). P value is indicated above the graph according to the Wilcoxon paired test.

To define whether endocytosis of NOTCH1 is necessary for N1‐ICD generation in CLL, we examined the effect of pitstop‐2, an inhibitor of early endocytic events.^45^, ^46^ To this end, N1‐ICD‐positive CLL cells were cultured for 30 minutes with 10 μM pitstop‐2 or DMSO as a control and analyzed by PLA to detect the colocalization of N1‐ECD, N1‐TM, and N1‐ICD with the EE marker Rab5. Figure 3A shows that Pitstop‐2 treatment reduced the percentage of PLA‐positive cells compared with control cells (Figures 3A and S7), indicating impaired early endocytic trafficking of NOTCH1. As a functional control for Pitstop‐2 activity, surface levels of CD71 (transferrin receptor) and transferrin were measured by flow cytometry. Pitstop‐2 treatment increased surface staining of both markers, consistent with inhibition of clathrin‐mediated internalization in CLL cells (Figure S8). To determine whether inhibition of NOTCH1 endocytosis affected N1‐ICD generation, we next analyzed the expression of N1‐ICD and N1‐TM by Western blot. Pitstop‐2 treatment reduced the levels of both NOTCH1 subunits compared with DMSO‐treated controls. Rab5 protein levels remained stable following treatment (Figure 3B), confirming that the reduced Rab5/NOTCH1 proximity observed by PLA was not due to changes in Rab5 expression.

Importantly, flow cytometry analysis demonstrated that Pitstop‐2 treatment increased surface NOTCH1 levels (Figure 3C), consistent with inhibition of endocytosis, and confirmed that the reduction of N1‐TM observed in total lysates reflects receptor redistribution rather than an overall decrease in NOTCH1 expression.

These data indicate that the reduction in N1‐ICD protein levels was due to impaired early events of NOTCH1 endocytosis caused by Pitstop‐2.

### Generation of N1‐ICD occurs in the endosomal membranes in CLL cells

**Figure 4:**
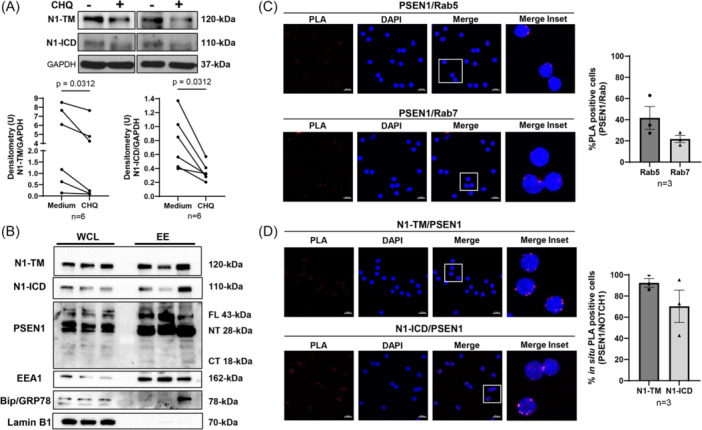
**Generation of N1‐ICD occurs in the endosomal membranes in chronic lymphocytic leukemia (CLL) cells**. **(A)** CLL cells were cultured for 5 h with 50 µM chloroquine (CHQ) or complete medium as a control. **(B)** Western blot analysis of N1‐TM and N1‐ICD. Upper panel, two representative CLL samples are shown. Bottom panel, dot and line diagrams of densitometry analysis of N1‐TM and N1‐ICD normalized to GAPDH (*n* = 6). P values are indicated above each graph according to the Wilcoxon paired test. **(B)** Western blot analysis of N1‐TM, N1‐ICD, and PSEN1 performed in whole‐cell lysates (WCL) and early endosome‐enriched fractions (EE) of CLL cells (*n* = 5). Analysis of PSEN1 shows the full‐length (FL) and the N‐terminal (NT) and C‐terminal (CT) fragments derived from PSEN1 proteolytic activation. Analysis of EEA1, a marker of EE, was performed to control the enrichment of the isolated EE fraction. Analysis of Lamin B1, a nuclear envelope marker, and of Bip/GRP78, an endoplasmic reticulum marker, was performed to exclude contaminations of EE with other cellular membranes and compartments relevant to Notch1 biosynthesis and processing. Three representative CLL samples are shown. **(C, D)** PLA was performed to detect the interactions of PSEN1 with Rab5 and Rab7 **(C)** and with N1‐TM and N1‐ICD **(D)**. Left, confocal microscopy images from one representative CLL sample. PLA signals indicating interactions are visualized by red spots and nuclei by DAPI staining. Images were acquired using confocal microscopy with a 63× oil immersion and 1.4 NA objective. Scale bar 10 µm. Right, bar graphs showing individual data points and mean ± SEM of the percentage of PLA‐positive cells in three CLL samples. For each patient, five fields per condition were analyzed and averaged to obtain a single value prior to statistical analysis.

It has been shown in other cell systems that activating NOTCH1 cleavages can occur in the limiting membranes of the endocytic compartment, where ADAMs and the γ‐secretase complex^23^ are located and activated by the acidic conditions of the vesicles.^47^, ^48^ Thus, we treated cells with CHQ, an endosomal acidification inhibitor known to raise endosomal pH and to inhibit proteolytic enzyme activity.^49^, ^50^ In N1‐ICD‐positive CLL cells, CHQ administration reduced the levels of both N1‐TM and N1‐ICD compared with control (Figure 4A) suggesting that acidification is critical for NOTCH1 cleavage in the endocytic compartment. This pattern suggests that CHQ predominantly affects γ‐secretase–dependent processing, leading to a stronger impact on N1‐ICD generation than on N1‐TM levels. LC3‐II accumulation confirmed the effectiveness of CHQ treatment (Figure S9). To investigate whether γ‐secretase responsible for N1‐TM cleavage is present and active within endocytic vesicles of CLL cells, we isolated EEs and analyzed the expression of N1‐TM, N1‐ICD, and PSEN1,^51^ the active subunit of the γ‐secretase complex. As shown in Figure 4B, both N1‐TM and N1‐ICD, as well as full‐length (FL) PSEN1, were enriched in EE fractions compared to whole‐cell lysates. In addition, EE fractions displayed the N‐terminal (NT) and CT fragments of PSEN1, indicating its proteolytic activation.^52^ Despite variable BiP‐positive ER contamination across samples, the absence of Lamin B1 and enrichment of EEA1 confirmed successful EE isolation.

To further support this finding, we examined the colocalization of Rab5, Rab7, and NOTCH1 subunits with PSEN1. As shown in Figures 4C and S10, PSEN1 colocalized with both Rab5 (41.7% ± 4.7% positive cells) and Rab7 (21.8% ± 2.6% positive cells), indicating that the enzyme is associated with both early and late endosomal markers.

Interestingly, we also found PSEN1 colocalized with N1‐TM (92.5% ± 3.4% positive cells) and N1‐ICD (70.3% ± 6.1% positive cells) (Figures 4D and S10). The higher proportion of PSEN1/N1‐TM‐positive cells relative to PSEN1/N1‐ICD‐positive cells is consistent with the role of N1‐TM as the substrate for PSEN1‐mediated cleavage.^23^, ^24^

These results suggested that the cleavage of NOTCH1 by PSEN1 occurred at the vesicular membranes, starting from EE. Altogether, these findings revealed that PSEN1 was active and capable of cleaving its substrate within endosomal compartments of N1‐ICD‐positive CLL cells.

### Rab5 and Rab7 are necessary for N1‐ICD generation in CLL cells

**Figure 5:**
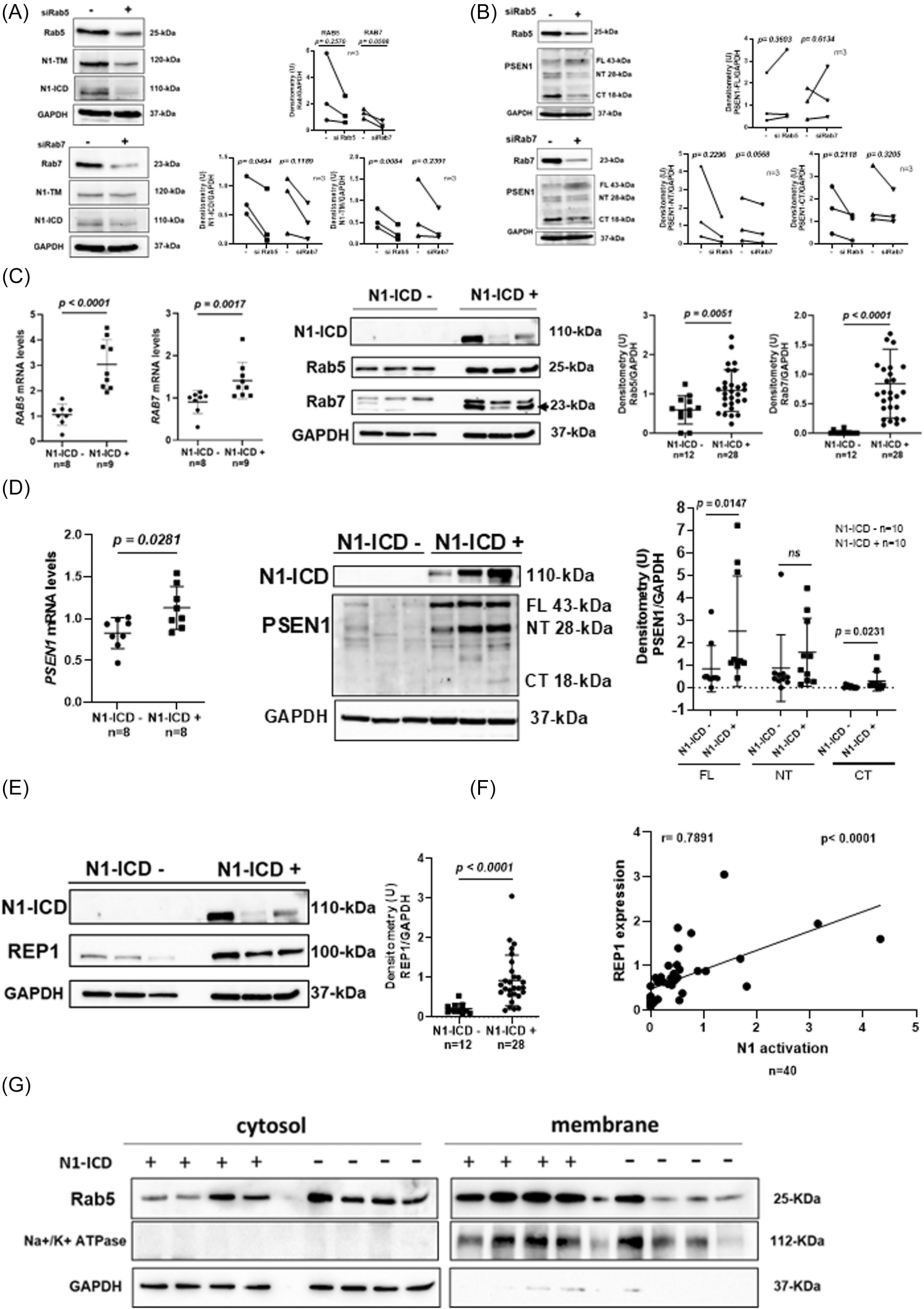
**Rab5 and Rab7 are required for NOTCH1 activation in chronic lymphocytic leukemia (CLL) cells**. **(A, B)** Left panels, representative Western blots of N1‐TM, N1‐ICD **(A)** and PSEN1 **(B)** from one N1‐ICD‐positive sample transfected with control siRNA, Rab5 siRNA (siRab5), or Rab7 siRNA (siRab7). Silencing efficiency was assessed by analyzing Rab5 or Rab7 expression. Analysis of PSEN1 shows the full‐length (FL) and the N‐terminal (NT) and the C‐terminal (CT) fragments derived from PSEN1 proteolytic activation. Protein loading was assessed by using an anti‐GAPDH antibody. Right panels, dot and line diagrams with connecting lines showing densitometry analysis of Rab5, Rab7, N1‐ICD, N1‐TM **(A)** and of PSEN1 full length (FL) and NT and CT cleaved fragments **(B)** in the three examined N1‐ICD‐positive samples. P values were calculated using a paired *t*‐test. **(C)** Analysis of Rab5, Rab7 expression was performed in N1‐ICD‐positive CLL cells and N1‐ICD‐negative CLL cells. Left panel, scatter dot plots with data points of real‐time polymerase chain reaction (PCR) analysis of *RAB5 and RAB7* mRNA levels normalized to GAPDH and represented as fold change using the mean of N1‐ICD‐negative cells as a reference. Middle panel, representative western blot data from three N1‐ICD‐negative and three N1‐ICD‐positive samples. Protein loading was assessed by using an anti‐GAPDH antibody. Right, scatter dot plots with data points of densitometry analysis of Rab5 and Rab7. Densitometry units (U) were calculated relative to GAPDH. P values were calculated according to the Mann–Whitney *U* test. **(D)** Analysis of PSEN1 expression was performed in N1‐ICD‐negative and N1‐ICD‐positive CLL cells. Left panel, scatter dot plots with data points of real‐time PCR analysis of *PSEN1* mRNA levels normalized to GAPDH and represented as fold change using mean of N1‐ICD‐negative cells as a reference. Middle panel, representative western blot data from three N1‐ICD‐negative and three N1‐ICD‐positive samples. Protein loading was assessed by using an anti‐GAPDH antibody. Right, scatter dot plots with data points of densitometry analysis of PSEN1 full length (FL) and NT and CT cleaved fragments. Densitometry units (U) were calculated relative to GAPDH. P values were calculated according to the Mann–Whitney *U* test. **(E)** Western blot analysis of REP1 was performed in N1‐ICD‐negative and N1‐ICD‐positive cells. Protein loading was assessed by using an anti‐GAPDH antibody. Left, representative CLL samples are shown. The same blot showed in **(C)** was used to evaluate REP1 expression in these samples, thus N1‐ICD and GAPDH images are repeated in this panel. Right, scatter dot plots with data points of densitometry analysis of REP1. Densitometry units (U) were calculated relative to GAPDH. P < 0.0001 according to the Mann–Whitney *U* test. **(F)** Correlation between N1‐ICD and REP1 densitometric values normalized to GAPDH, obtained from Western blot data shown in **(E)**, was assessed by using the Spearman's correlation coefficient (*r*). P < 0.0001. **(G)** Western blot analysis of Rab5 was performed in the cytosol and membrane‐enriched fraction isolated from N1‐ICD‐positive (*n* = 4) CLL cells and N1‐ICD‐negative (*n* = 4) CLL cells. The blots were reprobed with an anti‐GAPDH antibody to control the purity of membrane‐enriched fractions and the protein loading of cytosol, and with an anti‐Na+/K+ATPase antibody to control the purity of cytosol and the protein loading of membrane‐enriched fractions.

To better define the role of the endocytic compartment in the generation of active N1‐ICD, we downregulated the expression of Rab5 or Rab7 through siRNA in CLL cells. Cells were transfected with Rab5 (siRab5), Rab7 (siRab7), or nontargeting siRNA as control (siCtrl) and analyzed for the expression of Rab proteins, N1‐TM, N1‐ICD, and PSEN1 after 48‐hour culture. Upon silencing, we observed a reduced Rab5 and Rab7 expression associated with decreased levels of N1‐TM, N1‐ICD, and PSEN1 proteins compared to controls (Figure 5A,B). The reduction in PSEN1 NT and CT fragments was accompanied by a relative accumulation of the full‐length PSEN1 protein in two out of three analyzed patients. This pattern is consistent with the hypothesis that disruption of early or late endosome function through Rab5 or Rab7 silencing is likely to interfere with PSEN1 processing and activation rather than reduced PSEN1 expression. These data indicated that the deregulation of early and late endosomes impaired the machinery of N1‐ICD generation. In line with these observations, we found significantly higher expression levels of Rab5, Rab7, and PSEN1 in N1‐ICD‐positive compared to N1‐ICD‐negative CLL cells, both at the mRNA and protein level. Notably, Rab7 expression was almost undetectable in N1‐ICD‐negative samples (Figure 5C,D). Importantly, correlation analyses between N1‐ICD and Rab proteins confirmed a significant positive association between NOTCH1 activation and both Rab5 and Rab7 (Figure S11). Furthermore, PSEN1 NT and CT fragments were increased in N1‐ICD‐positive samples compared to N1‐ICD‐negative cases, indicating enhanced γ‐secretase activation (Figure 5D). In order to study whether Rabs overexpression was associated with increased activity, we measured the expression of the Rab escort protein 1 (REP1) in N1‐ICD‐positive compared to negative cells. REP1 is a key regulator of Rab activity that acts by presenting Rabs to the enzyme geranylgeranyl transferase II (GGTase II) for prenylation, a critical event for Rab translocation to membranes where Rabs become functional^32^, ^53^ (Figure S12). Figure 5E shows that N1‐ICD‐positive cells exhibited significantly higher levels of REP1 compared with N1‐ICD‐negative CLL. Furthermore, we found a positive correlation between REP1 and N1‐ICD levels (Figure 5F), as well as between REP1 and both Rab5 and Rab7 (Figure S11), further suggesting an association between Rab activity and NOTCH1 activation. Rab protein prenylation consists in the addition of two geranylgeranyl diphosphate groups to the CT cysteine of the Rabs^30^, ^31^, ^32^ (Figure S12). This event is essential so that Rabs can bind to lipid membranes and become functional, otherwise unprenylated Rabs remain in an inactive form in the cytosol. Based on this mechanism, we analyzed the distribution of Rab5 in cytosolic and membrane‐enriched fractions isolated from N1‐ICD‐positive and N1‐ICD‐negative cells. As shown in Figure 5G, Rab5 was predominantly enriched in the membrane fraction of N1‐ICD‐positive cells, whereas it accumulated mainly in the cytosol of N1‐ICD‐negative cells. These results further support a link between NOTCH1 activation and Rab5 membrane association and functionality. We next compared Rab expression in N1‐ICD‐positive CLL cells with that observed in B cells isolated from the peripheral blood of healthy donors (HD). CLL cells displayed significantly higher levels of Rab5 and Rab11 compared to HD B cells and a modest increase of Rab7 levels that did not reach statistical significance (Figure S13). These findings indicate that Rab‑dependent endocytic trafficking is quantitatively altered in CLL compared with healthy peripheral blood B cells, supporting the notion that this represents a CLL‐enhanced rather than a strictly CLL‐specific mechanism.

### Pharmacological targeting of Rab activity reduces N1‐ICD levels and CLL cell survival

To identify potential preclinical agents capable of modulating Rab activity, we targeted Rab prenylation by inhibiting the GGTase II enzyme^54^ in CLL cells using the natural compound psoromic acid (Figure S12).

**Figure 6:**
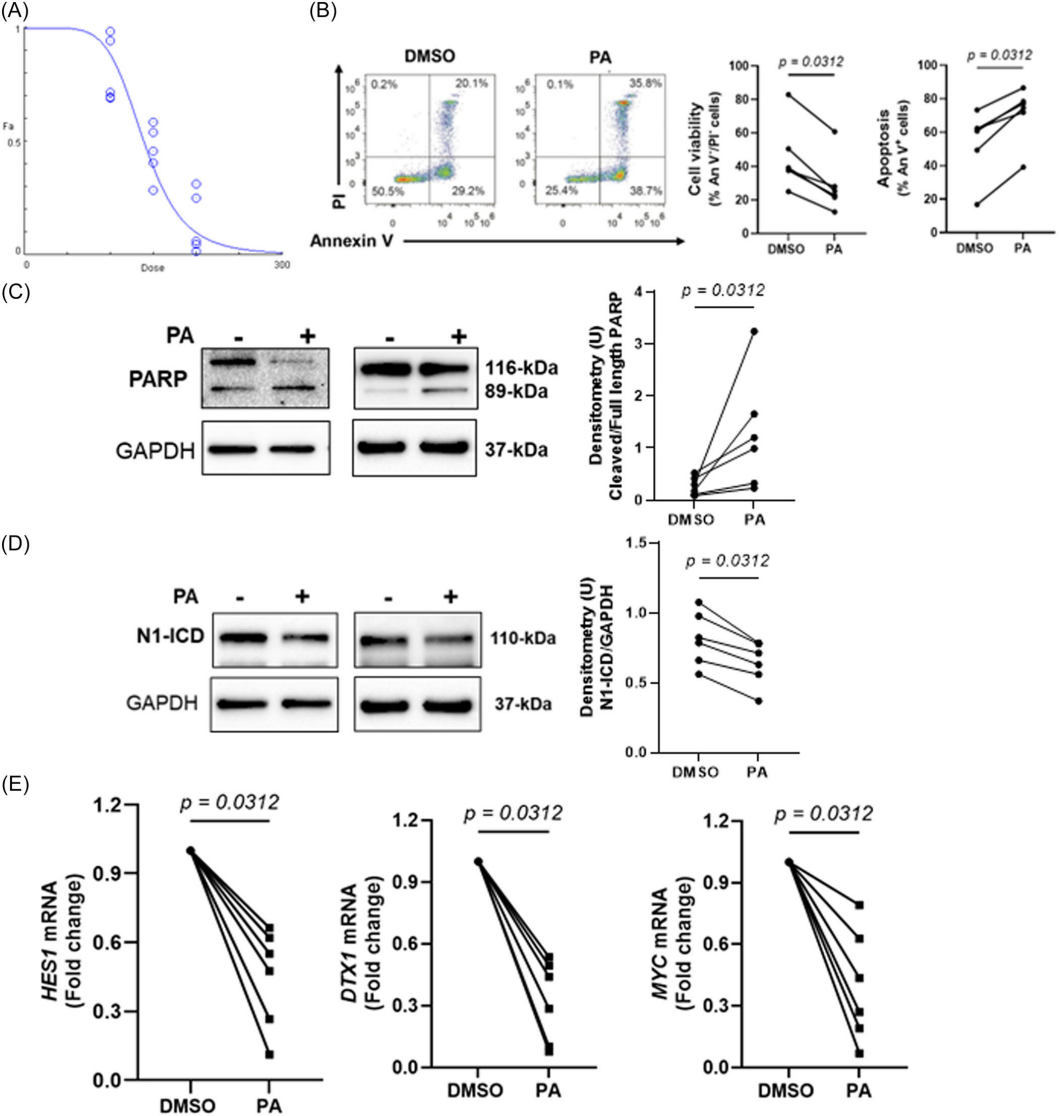
**Pharmacological inhibition of Rab activity reduces N1‐ICD levels and chronic lymphocytic leukemia (CLL) cell survival**. **(A)** Dose response curve of psoromic acid (PA) for primary CLL cells (*n* = 5) using CompuSyn software. **(B–E)** CLL cells were treated for 24 h with 140 µM PA or DMSO as a control (*n* = 6). **(B)** Cell viability and apoptosis were evaluated by flow cytometric analysis of Annexin V/PI (An V/PI) double staining. Results are represented as the percentage of viable (An V^−^/PI^−^), early apoptotic (An V^+^/PI^−^), late apoptotic (An V^+^/PI^+^), and necrotic (An V^−^/PI^+^) cells. Left, one representative CLL sample is shown. Middle and right, dot and line diagrams showing the percentage of viable An V^−^/PI^−^ (middle) and apoptotic An V^+^ (An V^+^/PI^−^ plus An V^+^/PI^+^) cells (right). P values are indicated above each graph according to the Wilcoxon paired test. **(C)** Western blot analysis of PARP cleavage. GAPDH was analyzed as a loading control. Left panel, two CLL samples are shown. Right panel, dot and line diagrams showing densitometry analysis of cleaved PARP (89‐kDa) normalized to levels of full‐length PARP (116‐kDa). **(D)** Western blot analysis of N1‐ICD levels. GAPDH was analyzed as a loading control. Left panel, two CLL samples are shown. Right panel, dot and line diagrams showing densitometry analysis of N1‐ICD normalized to GAPDH. For the sample on the right, the same blot shown in (**C**, right) was used; therefore, the GAPDH image is repeated in this panel. **(E)** Dot and line diagrams showing fold change in mRNA expression of NOTCH1 target genes (*HES1*, *DTX1*, and *c‐MYC*) in CLL cells treated with PA or DMSO. P values are indicated above each graph according to the Wilcoxon paired test.

We used the CellTiter‐Glo® assay to establish an IC_50_ value of 140 µM for PA in reducing CLL cells viability (Figure 6A). Figure 6B shows that PA reduced the percentage of viable AnV‐/PI‐cells and increased the percentage of early and late apoptotic AnV+ cells compared to DMSO. Consistent with these findings, PA treatment also led to enhanced PARP cleavage compared to DMSO (Figure 6C). Control membrane/cytosol fractionation confirmed reduced Rab membrane association upon PA treatment (Figure S14). Notably, these alterations in cell viability were associated with a reduction in N1‐ICD levels (Figure 6D) and NOTCH target genes (Figure 6E), underscoring the impact of Rab modulation on NOTCH1 activation.

### NOTCH1 trafficking is independent of *NOTCH1* mutational status

To assess whether *NOTCH1* mutational status affects receptor trafficking and processing, we extended our analyses to *NOTCH1*‐mutated CLL samples.

PLA analysis showed that N1‐ECD, N1‐TM, and N1‐ICD colocalized with Rab5 and Rab7, whereas Rab11 colocalized with N1‐ECD and N1‐TM and, to a lesser extent, with N1‐ICD (Figure S15A–C). This evidence together with the reduction in colocalization between NOTCH1 subunits and Rab5 observed in Pitstop‐2‐treated cells (Figure S16A) suggests that NOTCH1 undergoes endocytic trafficking through endosomal compartments also in the mutated context. Consistent with these observations and similarly to what observed with N1‐ICD‐positive wild‐type samples, inhibition of early endocytosis by Pitstop‐2 or blockade of endosomal acidification by CHQ led to a reduction in N1‐ICD generation in *NOTCH1*‐mutated cells (Figure S16B,C). In addition, PSEN1 colocalized with *NOTCH1* subunits and with Rab5‐ and Rab7‐positive compartments, supporting the involvement of endosomal γ‐secretase activity (Figure S17A,B). Indeed, silencing of Rab5 or Rab7 reduced N1‐TM and N1‐ICD and was also associated with decreased PSEN1 levels (Figure S18A), further supporting a functional link between endosomal trafficking and γ‐secretase activity. Pharmacological targeting of Rab activity with PA resulted in downregulation of N1‐ICD levels and apoptosis induction (Figure S18B).

Altogether, these findings indicate that *NOTCH1*‐mutated receptors remain dependent on endocytic trafficking and endosomal γ‐secretase activity for N1‐ICD generation, consistent with the mechanisms observed in NOTCH1 wild‐type CLL.

### NOTCH1 activation and Rab5 expression are associated with *IGHV* mutational status

Having established that NOTCH1‐mutated samples display trafficking and processing features comparable to wild‐type cases, we next investigated whether the NOTCH1–Rab axis is associated with established biological characteristics of CLL. To this end, we analyzed its relationship with *IGHV* mutational status, known to be associated with enhanced NOTCH1 activation.^43^

Patients were stratified into *IGHV*‐unmutated (*n* = 11) and *IGHV*‐mutated (*n* = 28) groups, and the expression levels of N1‐ICD, Rab proteins, and REP1 were compared between the two groups. The two NOTCH1‐mutated samples were excluded from this analysis to avoid potential bias. We found that N1‐ICD levels were significantly higher in *IGHV*‐unmutated cases compared to *IGHV*‐mutated ones. Similarly, Rab5 expression was significantly increased in *IGHV*‐unmutated CLL, whereas no significant differences were observed for Rab7, Rab11, or REP1 (Figure S19). These findings indicate that both NOTCH1 activation and Rab5 expression are enriched in the biologically aggressive *IGHV*‐unmutated subgroup.

## DISCUSSION

Ligand‐independent activation of NOTCH receptors within endosomal membranes has been well described in *Drosophila* cells, but whether this mechanism of NOTCH activation occurs in mammalian cells under physiological or oncogenic conditions remains unclear. In this respect, Steinbuck et al. demonstrated that TCR/CD28 signals induced endocytosis and consequent activation of NOTCH1 in the endosomal compartments^55^, ^56^ of murine T lymphocytes. However, whether the same mechanism contributes to ligand‐mediated activation of NOTCH1‐driven T leukemia cells is still unclear.

In CLL, several mechanisms of ligand‐independent NOTCH1 activation have been identified, including BCR engagement driven by microenvironmental signals,^57^ BTK activity,^58^ and IL‐4 stimulation.^21^ These findings further emphasize the critical role of NOTCH1 in CLL pathogenesis, but the molecular determinants and regulatory pathways underlying ligand‐independent NOTCH1 activation in this disease context remain largely unknown. A deeper understanding of these mechanisms could enable the identification of alternative therapeutic targets aimed at selectively inhibiting aberrant NOTCH1 signaling in leukemic cells, while sparing physiological, ligand‐dependent NOTCH1 functions in normal cells. Such a strategy may help overcome the limitations of current NOTCH1‐targeted therapies, whose clinical efficacy has been hampered by significant toxicity due to “on‐target, off‐tumor” effects.

In this study, we provided the first evidence that generation of the active N1‐ICD in CLL cells depends on NOTCH1 internalization and occurs at the membranes of the endocytic compartments, likely due to alterations in Rabs proteins and intracellular membrane‐bound enzymes responsible for NOTCH1 cleavage. Rabs are key regulators of the endocytic pathway.^27^, ^28^ Rab5 is a rate‐limiting catalyst of the endocytic uptake and delivery to the EEs, while Rab7 controls transport from EEs to late endosomes and from late endosomes to lysosomes, where NOTCH1 can be either activated at the vesicle membranes or degraded within their lumen. Conversely, Rab11 controls recycling to the plasma membrane.

Our PLA experiments show that both N1‐TM and N1‐ECD colocalize with Rab5, Rab7, and Rab11 markers in N1‐ICD‐positive CLL cells. These results indicate that the full‐length NOTCH1 receptor has been endocytosed into CLL cells and can be recycled to the plasma membrane, thus favoring interactions with NOTCH ligands expressed on microenvironmental cells into lymphoid tissues. Indeed, it is mainly in the lymph node niches that CLL cells exhibit the highest N1‐ICD levels, which contribute to cell proliferation and survival.^59^, ^60^

More importantly, our results showed that even N1‐ICD colocalizes with Rab5 and Rab7 markers, suggesting that the cleavage generating N1‐ICD could occur at the membranes of the endocytic vesicles. The proteolytic cleavages of NOTCH1 by the ADAM metalloproteases and the γ‐secretase complex represent the critical event for the release and nuclear translocation of N1‐ICD, with consequent signaling activation.^23^, ^24^ Notably, early and late endosomal structures not only control intracellular trafficking but also represent a compartment in which proteolytic events take place, including cleavages by the γ‐secretase complex.^61^

Here, we have found that the catalytic component of the γ‐secretase complex PSEN1^51^ is enriched in early endosomal compartments and shows partial colocalization with Rab5 and Rab7 expressing vesicular membranes in CLL cells.

Furthermore, we demonstrated the colocalization of PSEN1 with both N1‐TM and N1‐ICD, suggesting that the NOTCH1 cleavage occurs at the endocytic vesicle membranes level. It has been reported that other factors that facilitate the cleavage of endocytosed NOTCH1 in the endosomal membranes are the chemical changes occurring in these compartments, including Ca2+ depletion and the gradual acidification, which increases proteolytic activities.^47^, ^48^ Our results indicate that treatment with the endosomal acidification inhibitor CHQ reduced N1‐ICD and N1‐TM levels, demonstrating that endosome acidification contributes to NOTCH1 cleavages in CLL cells.

To further support that N1‐ICD is generated in the intracellular compartments of CLL cells, we showed that the inhibition of endocytosis elicited by pitstop‐2 reduced N1‐ICD protein levels and decreased the colocalization of N1‐TM and N1‐ECD subunits with Rab5, indicating that NOTCH1 endocytosis is required for its activation. Our flow cytometry results indicate that pitstop‐2 treatment blocks NOTCH1 internalization, resulting in the accumulation of intact receptor at the plasma membrane. This finding supports the interpretation that pitstop‐2 selectively interferes with NOTCH1 endocytosis and ICD generation without reducing total receptor expression, highlighting the role of endocytic trafficking in NOTCH1 signaling regulation. Another evidence supporting the involvement of the endocytic pathway in NOTCH1 activation in CLL cells is that downregulating Rab5 or Rab7 expression by siRNA transfection reduces N1‐ICD levels.

When we analyzed the colocalization of NOTCH1 subunits with Rab proteins in N1‐ICD‐negative CLL cells, we found that both N1‐ECD and N1‐TM colocalize with Rab5 similarly to what observed in N1‐ICD‐positive cells, indicating that NOTCH1 receptor is endocytosed even when it will not be activated. In contrast, the interactions of N1‐ECD and N1‐TM with Rab11 are lower in N1‐ICD‐negative than in N1‐ICD‐positive cells, indicating a reduced NOTCH1 recycling to the plasma membrane with the consequent reduced possibility of signaling activation in lymphoid tissues.

Strikingly, even N1‐TM/Rab7 interactions are lower in N1‐ICD‐negative than in N1‐ICD‐positive cells. Consistent with this finding, N1‐ICD‐negative cells display increased colocalization of NOTCH1 subunits with the lysosomal marker LAMP1, indicating preferential targeting toward lysosomal compartments. Collectively, these observations indicate that the early and late endocytic compartments exert an important regulatory control on NOTCH1 in CLL cells, determining signaling activation or inhibition. Notably, we observed a similar trafficking pattern in NOTCH1‐mutated samples, indicating that this regulatory mechanism is independent of NOTCH1 mutational status.

In an attempt to correlate different activation states of NOTCH1 with endosomal trafficking alterations in CLL, we found an increased expression of Rab5, Rab7, PSEN1, and REP1^32^, ^53^ (a key component of Rab activation) in N1‐ICD‐positive compared to negative cells together with increased PSEN1 fragments. Furthermore, we found a positive correlation between Rab5 and Rab7 with both N1‐ICD levels and REP1 expression. We also demonstrated that Rab5 was mainly located in the membrane‐enriched fraction rather than in the cytosol of N1‐ICD‐positive cells. Although NOTCH1 internalization occurs at comparable levels in both N1‐ICD‐positive and ‐negative CLL cells, we speculate that the key determinant lies in post‐endocytic trafficking rather than in the uptake step itself. In this context, Rab5 primarily regulates EE formation and maturation rather than the initial endocytic uptake, which is consistent with the similar levels of N1‐ECD association with Rab5 observed in both groups. Instead, differences in Rab5/Rab7‐dependent endosomal maturation, together with variations in the functional availability of the γ‐secretase complex, may regulate the residence time of NOTCH1 within permissive endosomal compartments. This, in turn, could influence whether the receptor undergoes productive cleavage leading to N1‐ICD generation or is instead directed toward lysosomal degradation.

In line with this, N1‐ICD‐positive CLL samples exhibited higher expression levels of Rab proteins compared to peripheral blood B cells from HD, indicating a generalized upregulation of the endocytic machinery in the leukemic context.

Notably, we observed that both NOTCH1 activation and Rab5 expression were enriched in *IGHV*‐unmutated CLL cases, a subgroup associated with more aggressive disease biology. This finding suggests that alterations in endocytic trafficking may accompany the higher NOTCH1 signaling activity observed in the *IGHV*‐unmutated subset.^43^ Given that *IGHV*‐unmutated CLL is characterized by increased responsiveness to microenvironmental stimuli, including BCR signaling, our data raise the possibility that Rab‐dependent trafficking may functionally integrate external signals with intracellular NOTCH1 activation.

These data were consistent with an upregulation of Rabs activity associated with N1‐ICD overexpression in CLL, suggesting that an altered endosomal system can trigger malignant NOTCH1 activation, once the receptor has been endocytosed. Indeed, mounting evidence has shown the involvement of endosomes and Rabs in cancer^62^, ^63^, ^64^ through the regulation of oncogenic pathways. For example, Rab2A promotes breast cancer stem cells and tumorigenesis by activating ERK signaling.^65^ Other studies showed that Rab27B controls palmitoylation‐dependent NRAS signaling in myeloid leukemia.^66^ Wang et al. demonstrated that Rab22a promotes the malignant phenotype of lung adenocarcinoma by upregulating the PI3K/Akt/mTOR pathway.^67^ To our knowledge, our study represented a first demonstration of the involvement of Rab alterations in CLL pathogenesis.

Based on our data and several other studies,^33^, ^34^, ^35^, ^36^, ^37^ Rabs may represent a promising target for cancer therapy. Despite this, the development of drugs against Rabs is lagging and problematic, mainly due to the high degree of homology among Rab family members and with other small GTPase families and to the incomplete knowledge of their regulation.^64^ One way whereby Rab activity can be inhibited is targeting their prenylation, and specifically the GGTase II. Indeed, this enzyme directly activates Rabs by catalyzing the addition of two geranylgeranyl groups to their CT cysteine residues, event which is crucial so that Rabs can bind to lipids membranes and become functional.^30^, ^31^, ^32^ In this study, we used the GGTase II inhibitor PA as a pharmacological tool to interfere with Rab prenylation. We found that PA reduces CLL cell viability by increasing apoptosis, and also reduces N1‐ICD levels and target genes. These results implied Rabs in CLL targeting treatment and encourage further studies to identify more selective Rab inhibitors.

**Figure 7:**
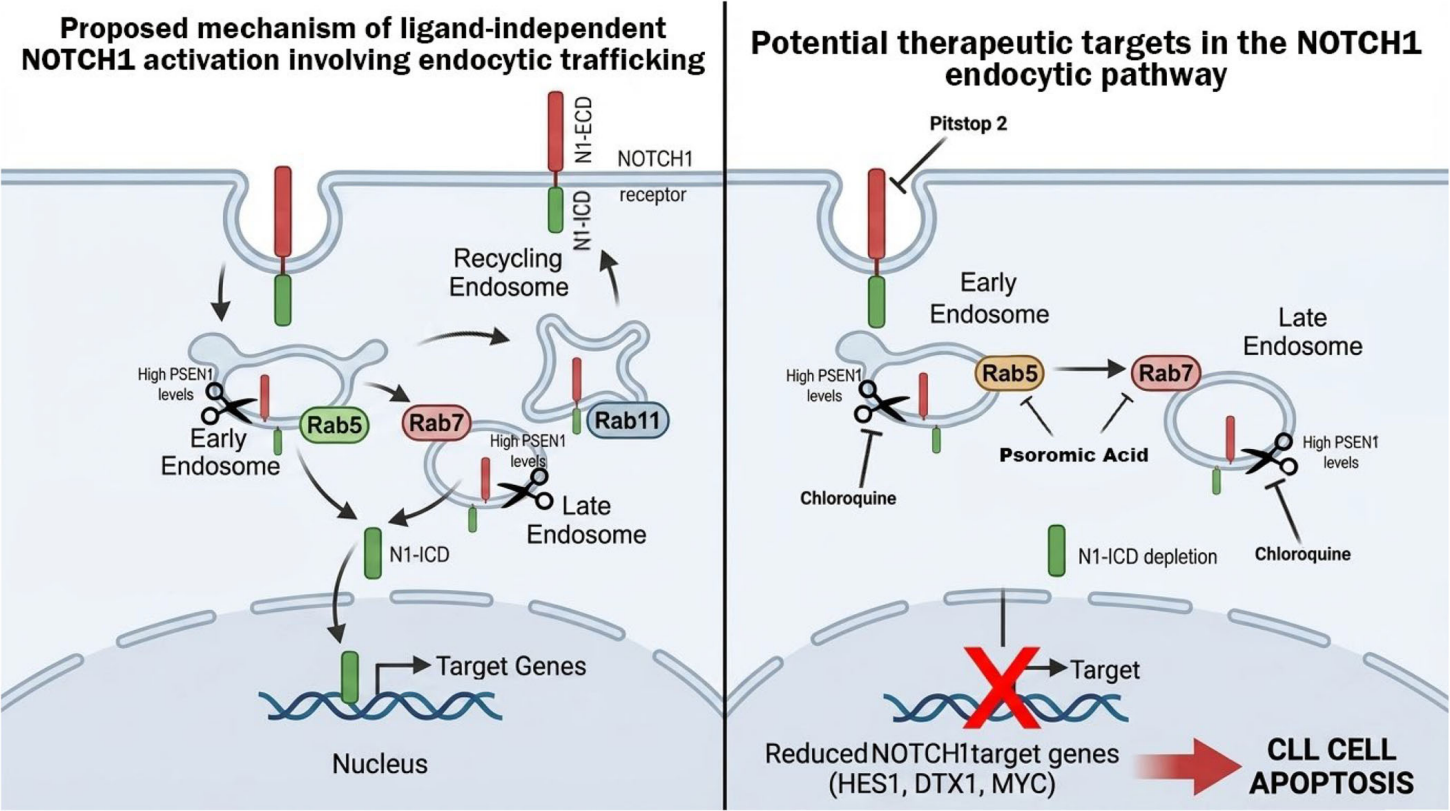
**Proposed mechanism of ligand‐independent NOTCH1 activation involving endocytic trafficking and potential therapeutic targets in chronic lymphocytic leukemia (CLL) cells**. In N1‐ICD‐positive CLL cells, the full‐length NOTCH1 receptor is internalized in early (Rab5) and late (Rab7) endocytic vesicles. PSEN1 on vesicular membranes cleaves NOTCH1 to generate the active N1‐ICD. The high levels of Rab5, Rab7, and PSEN1 expression/activity could be responsible for N1‐ICD generation in the membranes of the endocytic compartment (left panel). N1‐ICD levels can be reduced using pitstop‐2, chloroquine, or psoromic acid, which targets NOTCH1 endocytosis, PSEN1 activation in the endocytic compartments, and Rab function, respectively. Notably, psoromic acid reduces NOTCH1 target genes and also increases apoptosis in CLL cells (right panel).

Overall, we found in endosomal trafficking deregulation a novel intrinsic mechanism of NOTCH1 activation in CLL (Figure 7), which is independent of *NOTCH1* mutation and ligand interactions. We have also identified in Rab5 and Rab7 new regulators of NOTCH1 activation, which could be targeted for impairing NOTCH1 activation and reducing cell survival in CLL.

## AUTHOR CONTRIBUTIONS

**Filomena De Falco**: Data curation; investigation; formal analysis; visualization; writing—original draft. **Beatrice Del Papa**: Data curation; investigation; formal analysis. **Daniele Sorcini**: Investigation; visualization. **Letizia Valmarini**: Investigation. **Francesco Maria Adamo**: Investigation. **Andrea Atzeni**: Investigation. **Angela Esposito**: Investigation. **Estevão Carlos Silva Barcelos**: Investigation. **Erica Dorillo**: Investigation. **Arianna Stella**: Investigation. **Roberta Arcaleni**: Investigation. **Fabio Gurrieri**: Investigation. **Miriam Pugliese**: Investigation. **Gabriele Astolfi**: Investigation. **Maria Paola Martelli**: Writing—review and editing. **Mauro Di Ianni**: Writing—review and editing. **Emanuela Rosati**: Conceptualization; project administration; supervision; writing—original draft; visualization; methodology. **Paolo Sportoletti**: Project administration; supervision; funding acquisition; resources; writing—original draft; writing—review and editing.

## CONFLICT OF INTEREST STATEMENT

The authors declare no conflicts of interest.

## ETHICS STATEMENT

This study was approved by the local Ethics Committee, and all patients signed informed consent in accordance with the Declaration of Helsinki.

## FUNDING

This work was supported by the Associazione Italiana per la Ricerca sul Cancro (AIRC)—IG2024‐ID 30768 and IG2018‐ID 21352 to P.S.; by Gilead Sciences—Gilead Fellowship Program 2018 to P.S.; European Union—FSE‐REACT‐EU, PON Research and Innovation 2014–2020 DM1062/2021 to F.D.F.; FIRC‐AIRC (3‐years fellowship “Leonino Fontana and Maria Lionello” ID 26617 to F.M.A.); and University of Perugia “Fondo Ricerca di Ateneo, edizione 2021” to P.S. and E.R. Open access publishing facilitated by Università degli Studi di Perugia, as part of the Wiley ‐ CRUI‐CARE agreement.

## Supporting information

Supporting Information.

## Data Availability

The data that support the findings of this study are available from the corresponding author upon reasonable request.
