# Supplementary material for: Rab GTPases drive ligand‐independent NOTCH1 activation via altered endocytic trafficking in chronic lymphocytic leukemia
Source: Hemasphere. 2026 Jul 28;10(7):e70440. doi: 10.1002/hem3.70440 (PMC13410950; doi:10.1002/hem3.70440)
Supplement: Supplementary file 1 — Supporting Information. [file HEM3-10-e70440-s001.pdf]

Supplemental Information

Rab GTPases drive ligand-independent NOTCH1 activation via altered endocytic trafficking in Chronic Lymphocytic Leukemia

Contents:

- Supplemental Tables:

Supplemental Table 1. Characteristics of CLL patients

Supplemental Table 2. List of antibodies

Supplemental Table 3. Sequences of primers used for RT-PCR

Supplemental Table 4: Expression levels of N1-ECD, N1-TM and N1-ICD in CLL samples

- Supplemental Figures:

Supplemental Figure S1-S19

- Supplemental Methods:

Next-generation sequencing (NGS)

CLL co-culture with OP9-DLL1 stromal cells

Flow cytometry

Supplemental Table 1. Characteristics of CLL patients

Patient ID	Age	Sex	Rai/Binet stage	IGHV*	TP53	NOTCH1 mutational status (%)	Other NOTCH1 activating mutations (3'UTR, SF3B1, FBXW7, MED12)§	FISH †	% CD5/CD19
**CLL01**	57	M	II/B	Unm	Unm	Unm	SF3B1	no del11; no del17	94.9
**CLL02**	51	M	III/C	Unm	Unm	Unm	Absent	Nd	97.7
**CLL03**	50	F	0/A	Mut	Unm	Unm	Absent	del13	92.8
**CLL04**	66	M	0/A	Mut	Unm	Unm	Absent	del 13	94.5
**CLL05**	61	F	II/B	Mut	Unm	Unm	Absent	no del11; no del17	94.2
**CLL06**	55	F	0/A	Mut	Nd	Unm	Nd	del13	76
**CLL07**	71	F	0/A	Nd	Nd	Unm	Nd	Normal	84.1
**CLL08**	70	M	II/A	Mut	Unm	Unm	Absent	no del11; no del17	72.2
**CLL09**	50	M	I/A	Unm	Nd	Unm	Nd	del 13	86.6
**CLL10**	75	F	I/A	Mut	Unm	Unm	Absent	Normal	88.4
**CLL11**	65	M	II/B	Unm	Unm	Unm	MED12	no del11; no del17	88.2
**CLL12**	72	M	II/B	Unm	Unm	Unm	Nd	del13	95.5
**CLL13**	66	F	I/A	Mut	Nd	Unm	Nd	del13	99.2
**CLL14**	92	F	III/C	Mut	Nd	Unm	Nd	Normal	88.4
**CLL15**	61	F	II/B	Mut	Nd	Unm	Nd	del13	90.6
**CLL16**	84	M	IV/C	Mut	Unm	Unm	Absent	Normal	89.8
**CLL17**	59	F	I/B	Unm	Unm	Unm	Absent	Normal	93.1
**CLL18**	65	F	0/A	Mut	Unm	Unm	Absent	del13	97.3
**CLL19**	56	M	II/B	Mut	Unm	Unm	SF3B1	Normal	89.4
**CLL20**	52	M	IV/C	Mut	Unm	Unm	Absent	no del11; no del17	96.8
**CLL21**	69	F	II/B	Mut	Mut	Unm	Nd	del11	85.4
**CLL22**	80	F		Mut	Mut	Unm	Absent	Nd	79.8
**CLL23**	60	F	0/A	Mut	Unm	Unm	Absent	del13	82.24
**CLL24**	71	M	IV/C	Unm	Unm	Unm	Absent	del11	72.17
**CLL25**	72	M	I/A	Mut	Unm	Unm	SF3B1	Normal	84.73
**CLL26**	57	M	0/A	Unm	Unm	Unm	Absent	Normal	85
**CLL27**	74	M	0/A	Mut	Unm	Unm	Absent	Normal	79.3
**CLL28**	85	M	I/A	Mut	Unm	Unm	Absent	Nd	97.9
**CLL29**	56	M	II/A	Mut	Unm	Unm	Absent	Normal	80.3
**CLL30**	73	M	II/A	Mut	Unm	Unm	Absent	del13	80.4
**CLL31**	59	M	0/A	Mut	Unm	Unm	Nd	del13	87.2
**CLL32**	81	M	IV/C	Mut	Mut	Unm	Absent	no del11; no del17	94.9
**CLL33**	60	M	0/A	Mut	Unm	Unm	Absent	no del11; no del17	71.11
**CLL34**	80	F	III/A	Mut	Unm	Unm	Absent	Normal	81.2
**CLL35**	68	M	II/B	Mut	Unm	Unm	Absent	Normal	97.7
**CLL36**	52	F	II/B	Mut	Unm	Unm	Absent	no del11; no del17	95.2
**CLL37**	64	M	IV/C	Unm	Unm	Unm	Absent	no del11; no del17	94.8
**CLL38**	78	M	IV/C	Mut	Unm	Unm	Absent	Nd	82.5
**CLL39**	40	M	II/B	Unm	Unm	Unm	Nd	tris12	Nd
**CLL40**	70	F	III/C	Unm	Unm	Unm	Absent	del11	90.3

**CLL41**	53	M	II/B	Unm	Unm	Mut (46%)	Absent	no del11; no del17	79
**CLL42**	81	F	II/B	Unm	Unm	Mut (44%)	Absent	no del11; no del17	94

Abbreviations: M, male; F, female; Mut, mutated; Unm, unmutated; nd, not determined;

[*] Mutated was defined as having a frequency of mutations >2% from germline *VH*.
[†]Assessed by FISH.
[§]Assessed by NGS.

Supplemental Table 2. List of antibodies

Target protein	Clone	Species	Supplier	Catalog number	Application
NOTCH1-ICD (Val1744)	D3B8	Rabbit	CST	#4147	WB<br>PLA
NOTCH1-TM	D1E11	Rabbit	CST	#3608	WB<br>PLA
NOTCH1-ECD	ABS90	Rabbit	EMD Millipore	N/A	PLA
NOTCH1-PE	527425		R&D System	FAB5317P	FC<br>(Pitstop-2 treated cells)
NOTCH1-APC	22E5		Thermo Fisher	17-5765-82	FC
BIM	C34C5	Rabbit	CST	2933	PLA
BCL2	124	Mouse	Dako	M0887	PLA
Rab 5	D-11	Mouse	SCB	sc-46692	WB<br>PLA
Rab 7	B-3	Mouse	SCB	sc-376362	WB<br>PLA
Rab 11	A-6	Mouse	SCB	sc-166912	PLA
LAMP1	25/Lamp-1	Mouse	BD Transduction	611042	PLA
CD71-APC-AF700	YDJ1.2.2		Beckman Coulter	A97051	FC
LC3B	Polyclonal	Rabbit	Novus	NB600-1384	WB
Presenilin 1	APS 18	Mouse	Thermo Fisher	MA1-752	WB<br>PLA
Presenilin 1	Polyclonal	Rabbit	Thermo Fisher	PA5-30585	PLA
Lamin B1	Polyclonal	Rabbit	Abcam	ab16048	WB
BiP/GRP78	40/BiP	Mouse	BD Transduction	610979	WB
EEA1	EPR4245	Rabbit	Abcam	ab109110	WB
Na+/K+ ATPasi	Polyclonal	Rabbit	Proteintech	14418-1AP	WB
REP1	Polyclonal	Rabbit	Sigma	HPA003231	WB
β-Actin	AC-15	Human/<br>Mouse	Novus	NB 600-501	WB
GAPDH	GAPDH-71.1	Mouse	Sigma	G8795	WB

Abbreviations: CST, Cell Signaling Technology; SCB: Santa Cruz Biotechnology; WB, western blot; PLA, proximity ligation assay; FC, flow cytometry

Supplemental Table 3. Sequences of primers used for RT-PCR

Gene name	Forward primer (5' to 3')	Reverse primer (5' to 3')
RAB5	GGAGAGTCCGCTGTTGGCAAA	GGTGCTAGGCTATGGTATCGTTC
RAB7	CACAATAGGAGCTGACTTTCTGACC	GTTCCTGTCCTGCTGTGTCCCATATC
PSEN1	GTGTGGTTGGTGAATATGGCA	GCCCTAGATGACTGTCCCTC
HES1	AAGAAAGATAGCTCGCGGCAT	CCAGCACACTTGGGTCTGT
DTX1	CAGCCGCCTGGGAAGATGGAGTT	TGGATGCCTGTGGGGATGTCATAGAC
c-MYC	AATGAAAAGGCCCCCAAGGTAGTTATCC	GTCGTTTCCGCAACAAGTCCTCTTC
GAPDH	ATGGGGAAGGTGAAGGTCG	GGGGTCATTGATGGCAACAATA

Supplemental Table 4: Expression levels of N1-ECD, N1-TM and N1-ICD in CLL samples

Patients	N1-ECD levels	N1-TM levels	N1-ICD levels
**CLL1**	18.4%	3.23	1.69
**CLL2**	12.1%	1.84	0.51
**CLL3**	18.8%	1.64	0.24
**CLL4**	20.6%	1.50	0.40
**CLL5**	18.7%	1.87	0.54
**CLL6**	8.72%	1.86	0.09
**CLL7**	19.8%	1.67	0.44
**CLL8**	12.4%	2.10	0.50
**CLL9**	10.5%	1.91	1.04
**CLL10**	38.2%	0.15	0.14
**CLL11**	11.4%	0.39	1.81
**CLL12**	35.9%	0.08	0.06
**CLL13**	22.4%	0.02	0.03
**CLL14**	19.3%	0.21	0.14
**CLL15**	16.8%	0.35	0.47
**CLL16**	15.9%	ND	0.54
**CLL17**	19.4%	1.67	0.89
**CLL18**	34.5%	1.15	0.34
**CLL19**	21.2%	0.96	0.60
**CLL20**	7.68%	ND	0.20
**CLL21**	16.1%	ND	0.34
**CLL22**	18.3%	0.89	0
**CLL23**	8.08%	1.12	0
**CLL24**	22.1%	0.53	0
**CLL25**	16.6%	1.00	0
**CLL26**	13.4%	0.03	0

**CLL27**	14.5%	1.50	0
**CLL28**	24.4%	1.03	0
**CLL29**	16.8%	0.89	0
**CLL30**	15.3%	0.84	0
**CLL31**	ND	0.71	0
**CLL32**	12.1%	ND	0
**CLL33**	12.4%	ND	0
**CLL34**	29.5%	1.43	3.15
**CLL35**	15.8%	0.19	0.38
**CLL 36**	21.4%	2.60	4.33
**CLL37**	26.2%	0.19	1.39
**CLL38**	17.6%	0.10	0.51
**CLL39**	ND	0.16	0.76
**CLL40**	21%	0.07	0.52
**CLL41**	21.4%	ND	1.88
**CLL42**	25.3%	ND	1.53

Abbreviations: ND, not determined.
N1-ECD levels were determined by surface staining using the NOTCH1 (22E5) APC antibody and flow cytometry analysis. N1-TM and N1-ICD levels were expressed as a ratio between N1 to GAPDH levels evaluated by Western blot analysis using the N1-D1E11 and N1-D3B8 (Val1744) antibody respectively.

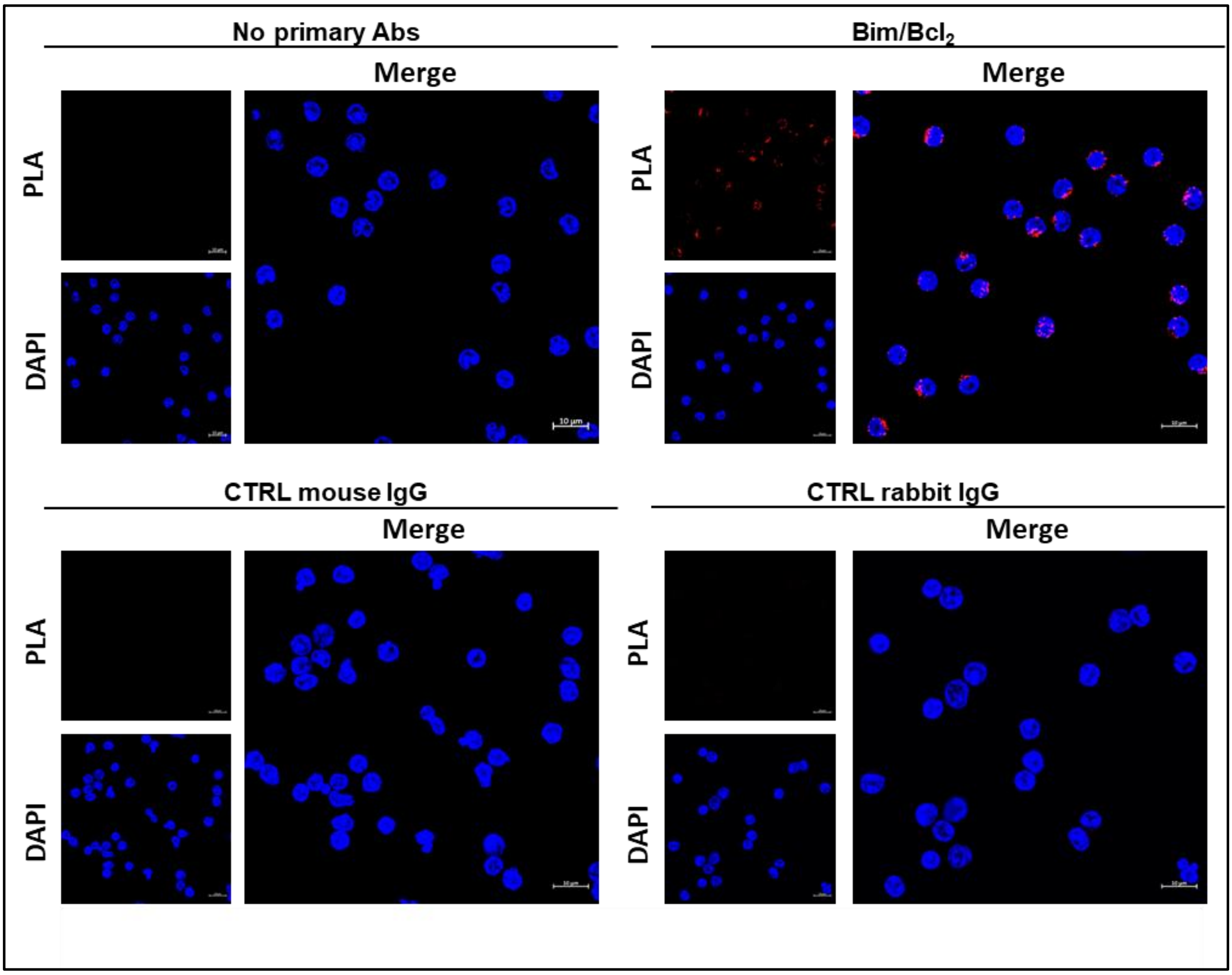

Supplemental Figure 1. Proximity ligation assay controls. Negative controls were obtained by omitting primary antibodies (Abs) or using control isotype antibody. Positive controls were obtained analyzing Bcl2-Bim colocalization, as a known protein-protein interaction. One representative field per condition is shown.

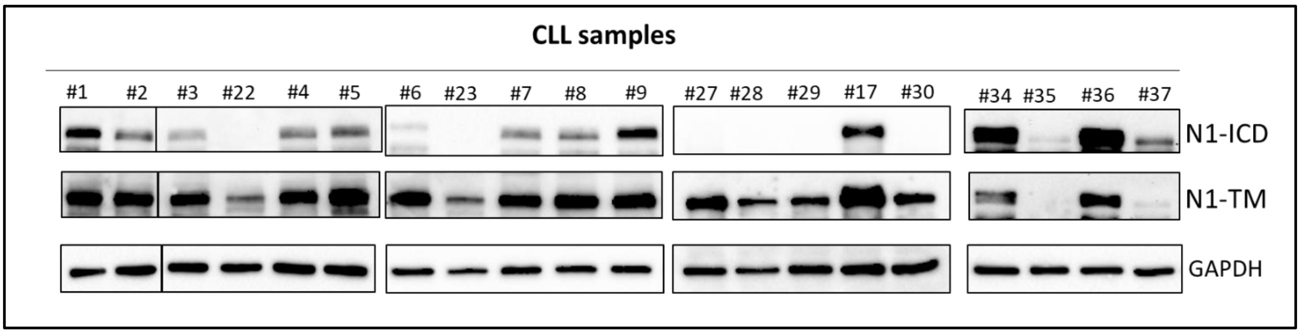

Supplemental Figure 2. Expression levels of N1-ICD and N1-TM in CLL patients. Western blot analysis of N1-ICD and N1-TM in whole cell lysates from NOTCH1-WT primary CLL cells (n = 40). Protein loading was assessed reprobing the blots with an anti-GAPDH antibody. Twenty-two representative samples are shown.

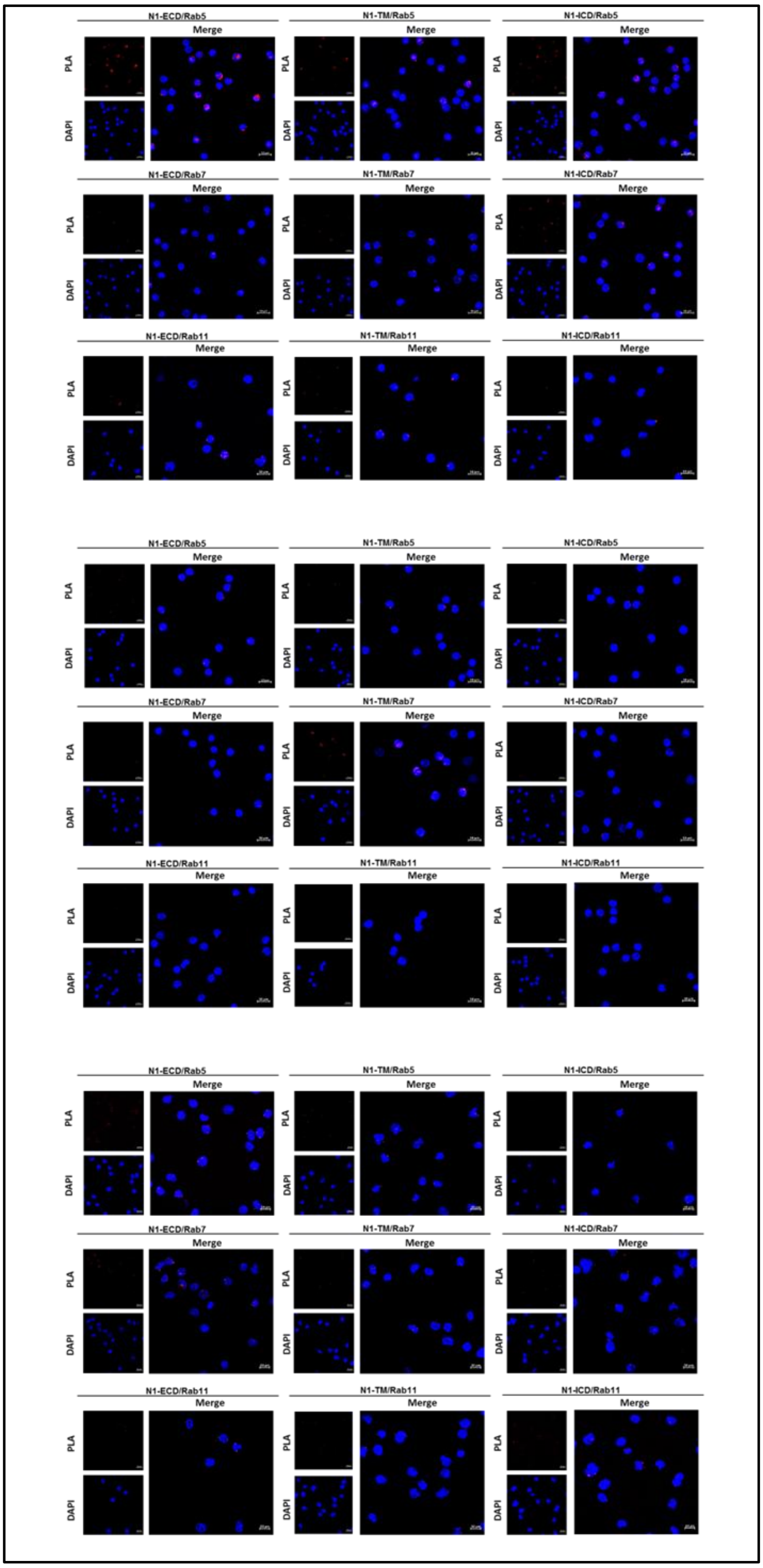

Supplemental Figure 3. NOTCH1 colocalization with Rab5, Rab7 and Rab11 markers in N1-ICD positive cells. Representative confocal microscopy images from three independent CLL samples analyzed by PLA are shown. Additional images not included in Figure 1 are presented here.

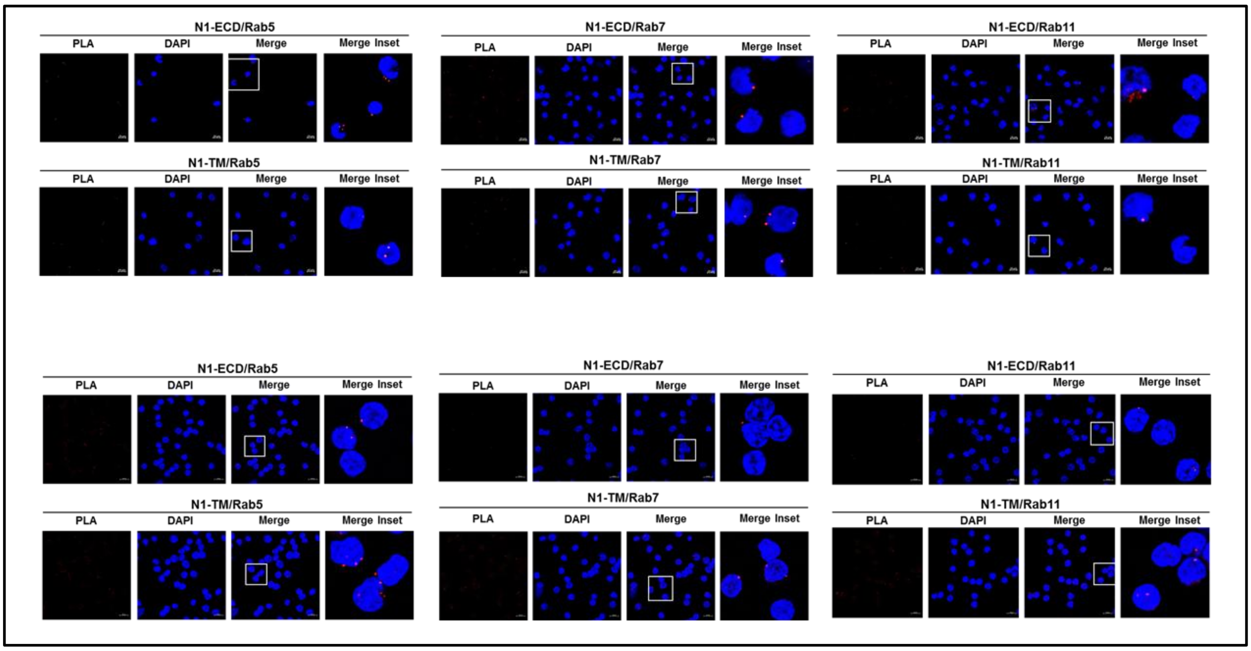

Supplemental Figure 4. NOTCH1 colocalization with Rab5, Rab7 and Rab11 markers in N1-ICD negative cells. Representative confocal microscopy images from two independent CLL samples analyzed by PLA are shown. Additional images not included in Figure 2 A-C are presented here.

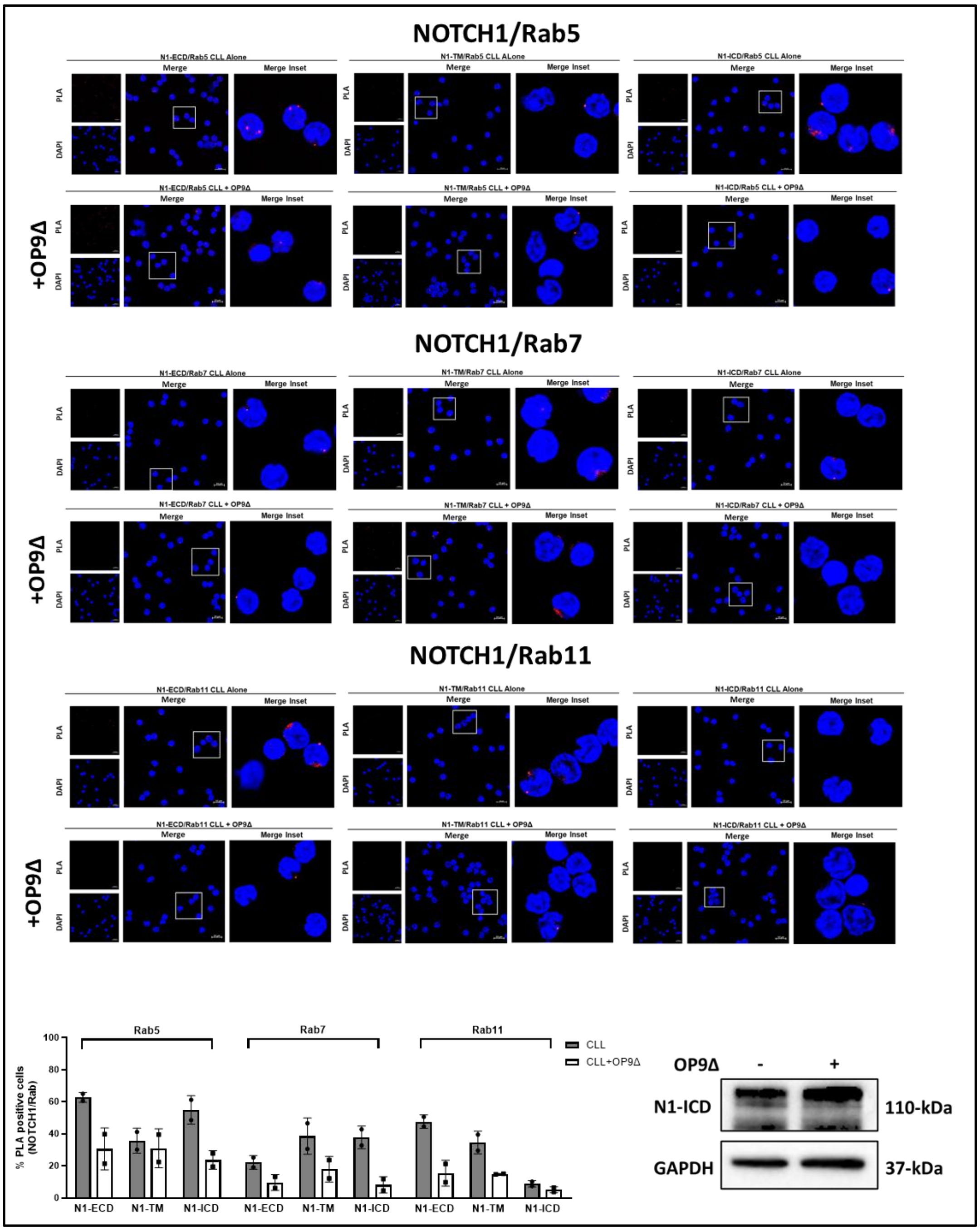

Supplemental Figure 5. Analysis of NOTCH1 colocalization with Rab5, Rab7 and Rab11 markers in N1-ICD positive cells co-cultured with OP9 cells expressing DLL1 ligand. PLA was performed in N1-ICD positive CLL cells alone or co-cultured with OP9 DLL1(OP9Δ) to detect the interaction of N1-ECD, N1-TM and N1-ICD with Rab5 (A), Rab7 (B) and Rab11 (C). Upper panels, confocal microscopy images for each interaction from one representative CLL sample are shown. PLA positive signals are visualized by red spots and nuclei by DAPI staining. Images were acquired using confocal microscopy with a 63x oil immersion and 1.4 NA objective. Scale bar 10μM. Bottom left panel, bar graphs with individual data points showing mean±SEM of the percentage of PLA positive cells for each interaction in CLL cultured alone or co-cultured with OP9Δ (n=2). Bottom right panel, WB analysis of N1-ICD levels confirming ligand-induced Notch1 activation.

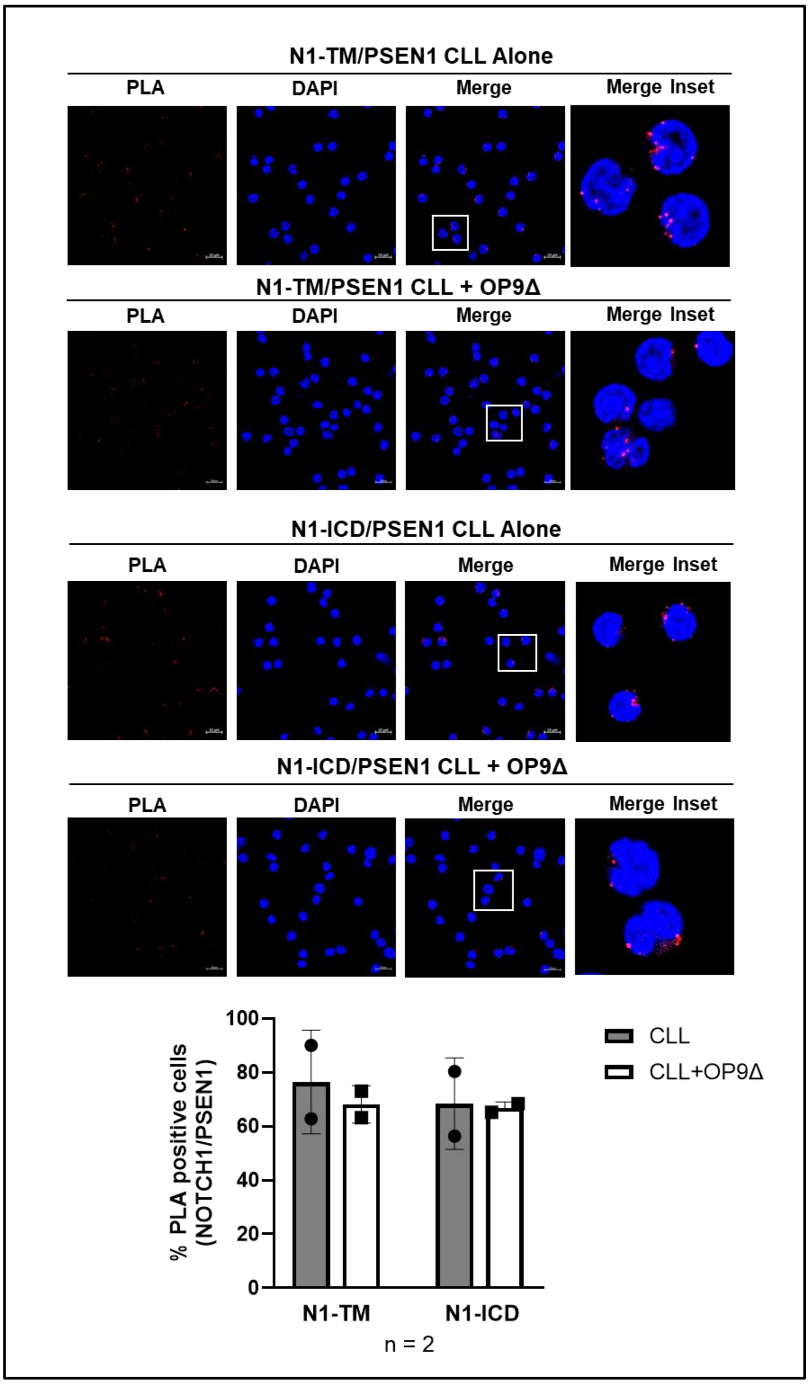

Supplemental Figure 6. Analysis of N1-TM and N1-ICD colocalization with PSEN1 in N1-ICD positive cells co-cultured with OP9 cells expressing DLL1 ligand. PLA performed in N1-ICD positive CLL cells alone or co-cultured with OP9 DLL1(OP9Δ) to detect the interactions of PSEN1 with N1-TM (upper panel) and N1-ICD (middle panel). Confocal microscopy images from one representative CLL sample are shown. PLA signals indicating interactions are visualized by red spots and nuclei by DAPI staining. Images were acquired using confocal microscopy with a 63x oil immersion and 1.4 NA objective. Scale bar 10 μm. Bottom panel, bar graphs showing individual data points showing mean±SEM of the percentage of PLA positive cells in two CLL samples.

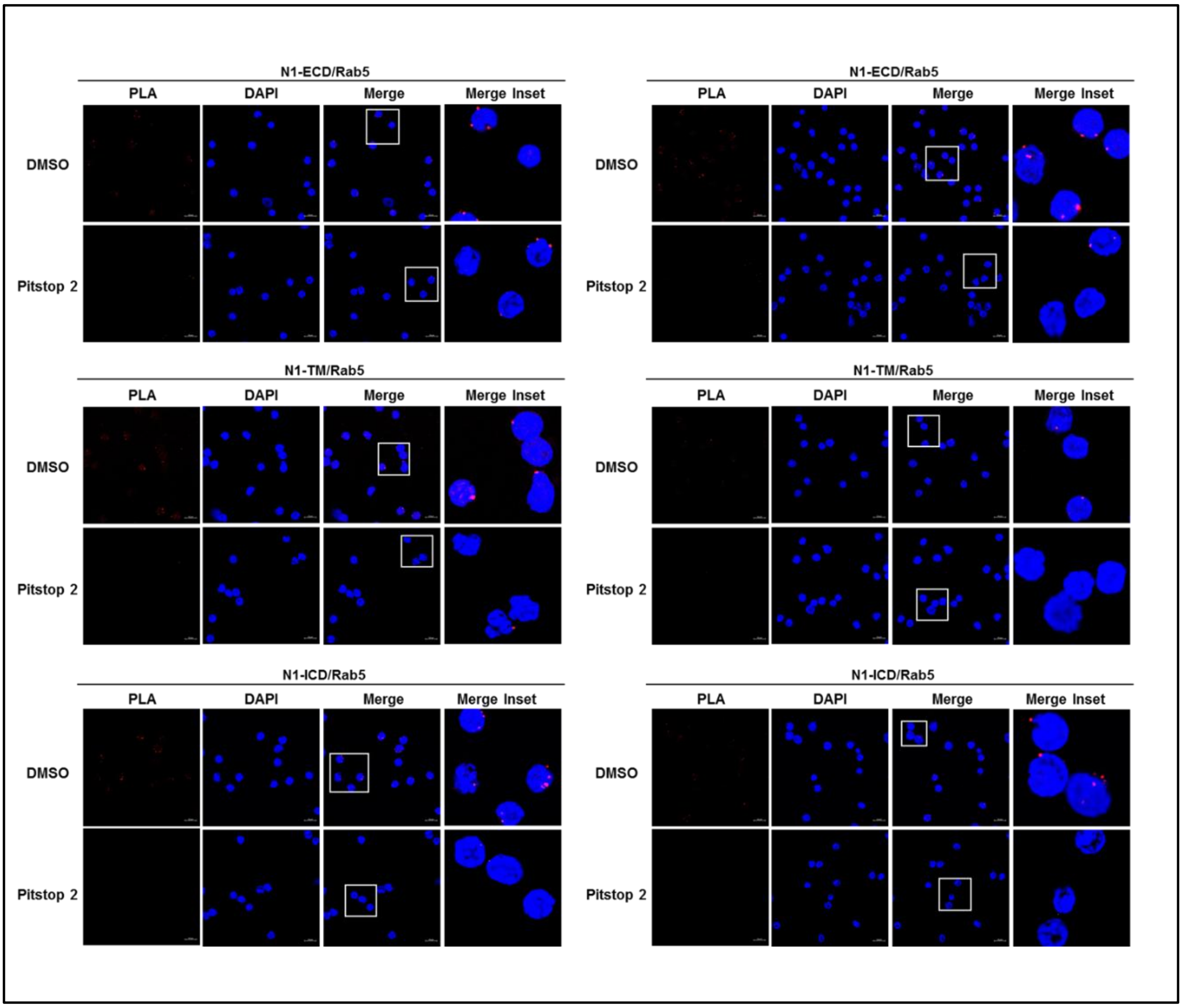

Supplemental Figure 7. NOTCH1 subunits colocalization with the early endosome marker Rab5 after endocytosis inhibition by Pitstop2. Representative confocal microscopy images from two independent CLL samples analyzed by PLA are shown. Additional images not included in Figure 3A are presented here.

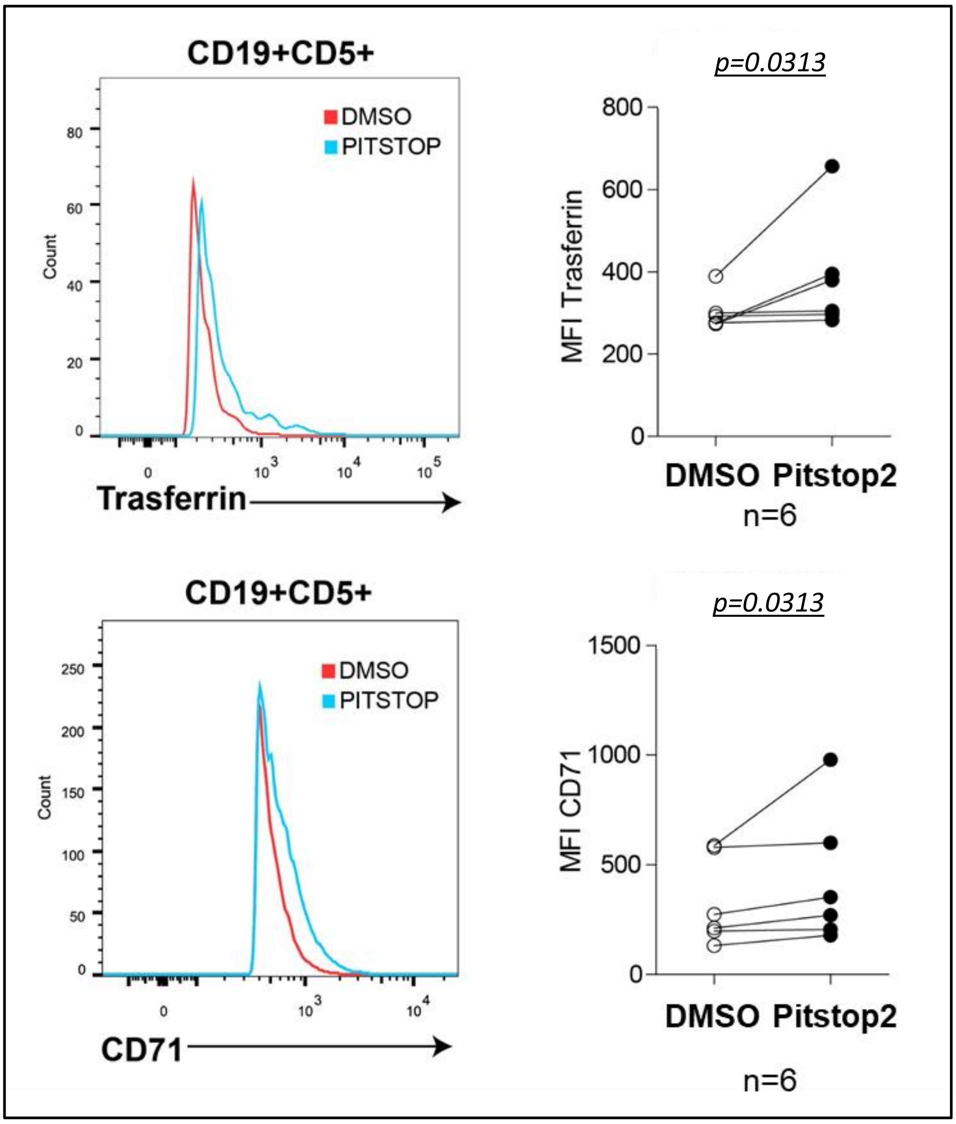

Supplemental Figure 8. Surface staining of Transferrin and CD71 in Pitstop-2 treated CLL cells. Analysis of surface transferrin (upper panel), performed in CLL cells (n=6) incubated with Alexa Fluor 488–conjugated transferrin (Thermo Fisher Scientific) at 37°C for 30 minutes in the presence or absence of Pitstop-2 before flow cytometry analysis. Bottom panel, CD71 surface staining of CLL cells (n=6) treated with Pitstop-2 or DMSO as control for 30 minutes before flow cytometry analysis. Representative plots and dot and line diagrams of Mean Fluorescence Intensity (MFI) are shown. P values are indicated above each graph according to Wilcoxon paired test.

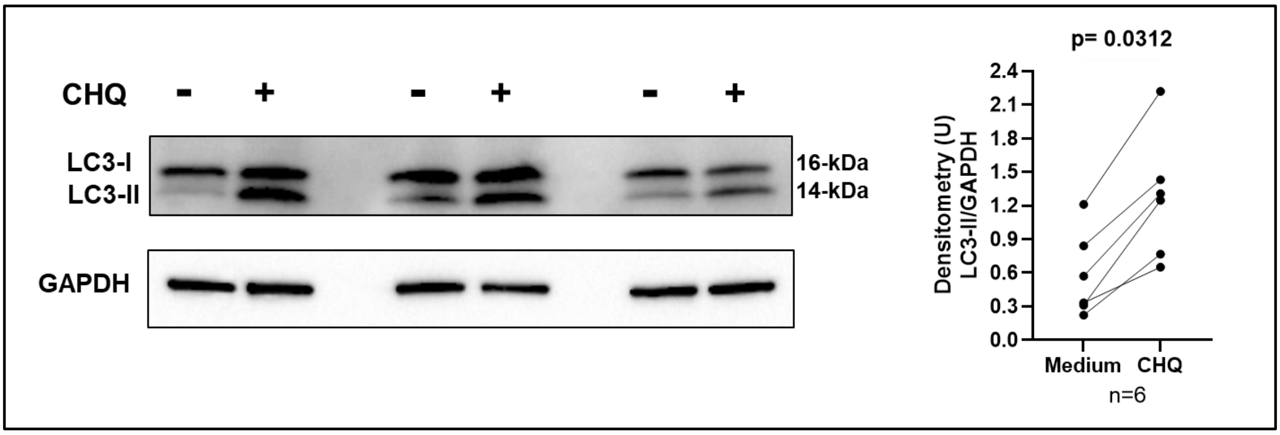

Supplemental Figure 9. Chloroquine effects on LC3-II accumulation. CLL cells were cultured for 5 hours with 50µM chloroquine (CHQ) or complete medium as control and LC3-II accumulation was analyzed by Western blot using GAPDH as loading control. Left panel, three representative CLL samples are shown. Right panel, paired dot plots showing densitometric analysis of LC3-II normalized to GAPDH (n = 6). P values is indicated above the graph according to Wilcoxon paired test.

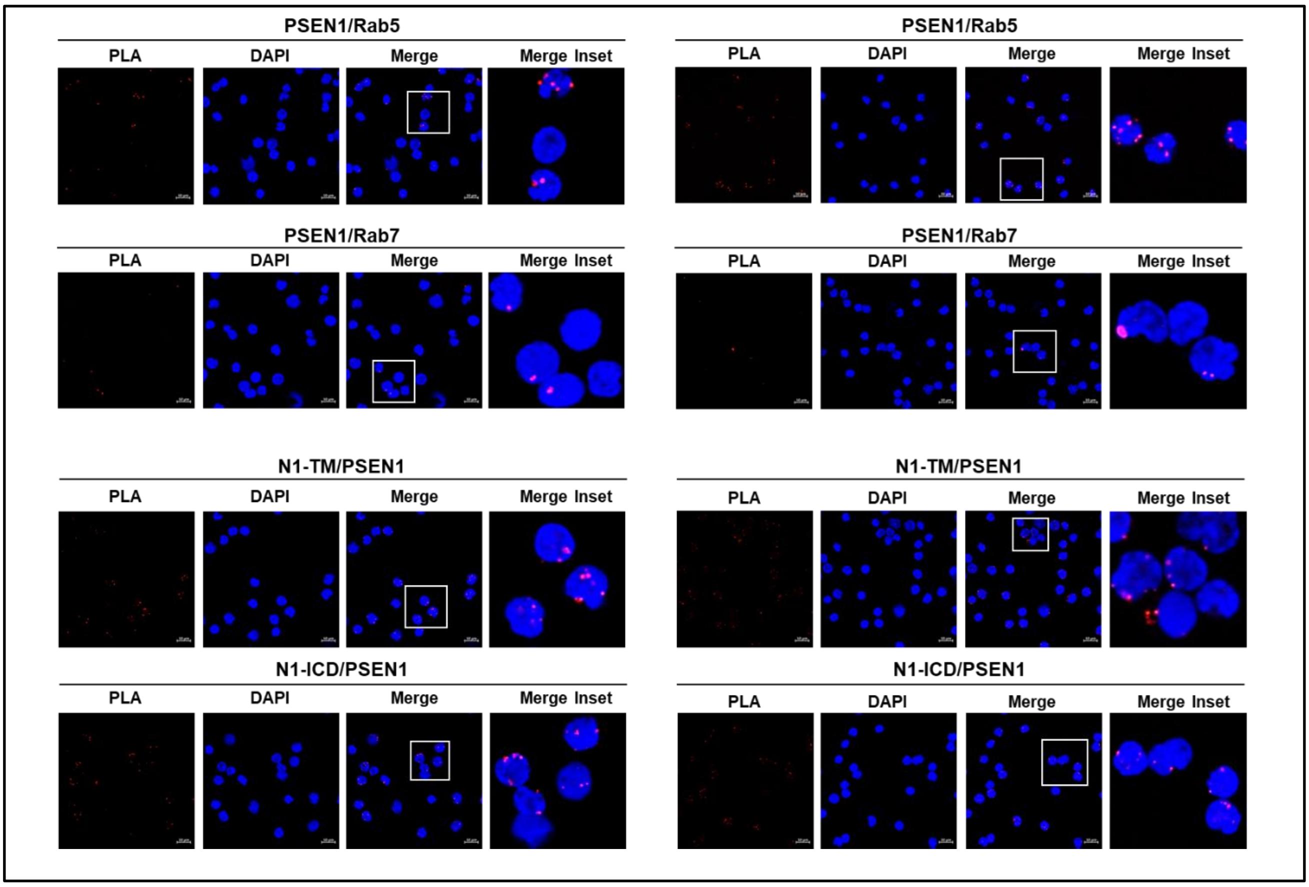

Supplemental Figure 10. Colocalization of PSEN1 with Rab5 and Rab7 and with N1-TM and N1-ICD in N1-ICD positive cells. Representative confocal microscopy images from two CLL sample analyzed but not shown in Figure 4C-D. PLA signals indicating interactions are visualized by red spots and nuclei by DAPI staining. Images were acquired using confocal microscopy with a 63x oil immersion and 1.4 NA objective. Scale bar 10µM

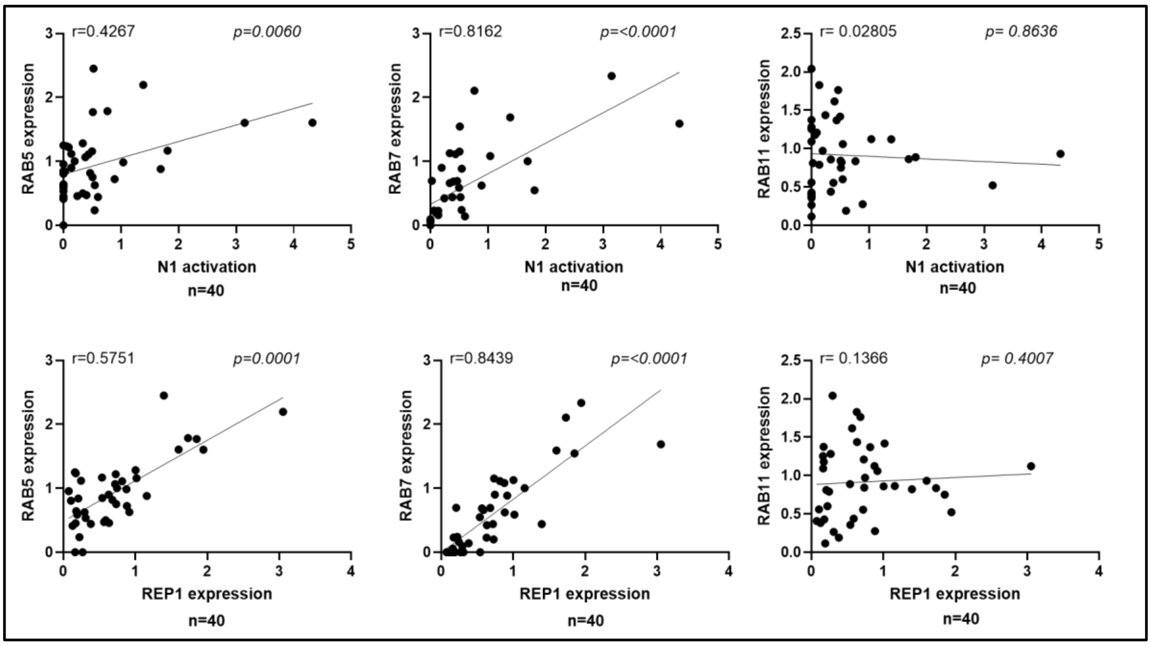

Supplemental Figure 11. Correlation analysis of RAB protein levels with N1 activation and REP1 expression. Correlation analysis of Western blot data in our patient cohort using densitometric values normalized to GAPDH was obtained by using the Spearman's correlation coefficient (r). P values are indicated above each graph.

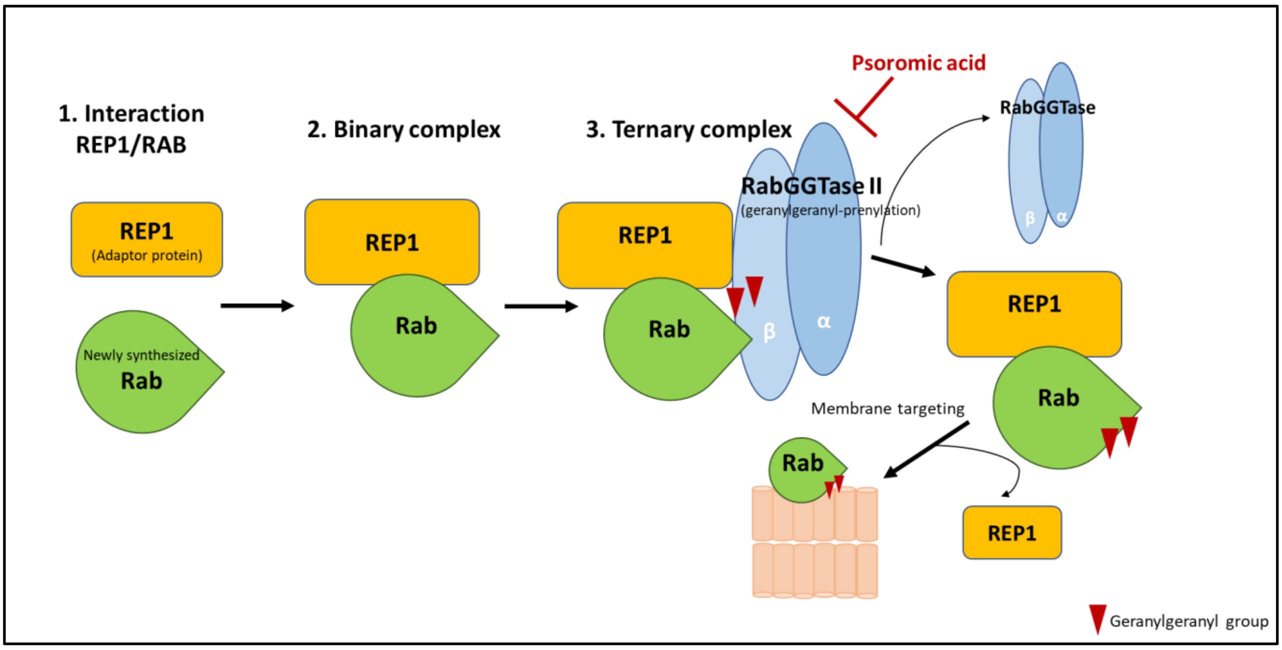

Supplemental Figure 12. Schematic representation of Rab protein prenylation. The diagram illustrates the key steps of Rab protein prenylation, a post-translational modification essential for Rab membrane association and function. Newly synthesized Rab proteins in the cytosol are first recognized by Rab escort protein 1 (REP1), which delivers them to the Rab geranylgeranyl transferase II complex (RabGGTase II). RabGGTase II catalyzes the covalent attachment of one or two geranylgeranyl groups to conserved cysteine residues at the C-terminus of Rab proteins. The prenylated Rab is then transferred back to REP1 and subsequently delivered to its target membrane, where it performs its role in vesicle trafficking. This process is crucial for proper Rab localization and activity within the endosomal system.

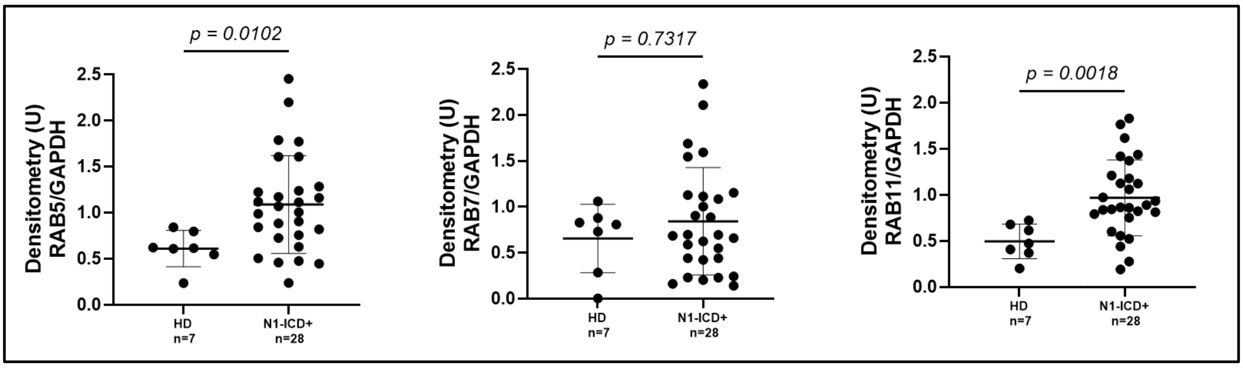

Supplemental Figure 13. Rab protein expression in N1-ICD positive CLL cells versus healthy donor B cells. Dot plots showing the distribution of RAB5, RAB7, RAB11 expression levels in N1-ICD positive CLL samples (n=28) compared to healthy donor peripheral blood B cells (n=7). Each dot represents an individual patient value calculated as a protein to GAPDH ratio and expressed as densitometric units (U), Statistical analysis was performed using the Mann–Whitney test; p value is indicated above each graph.

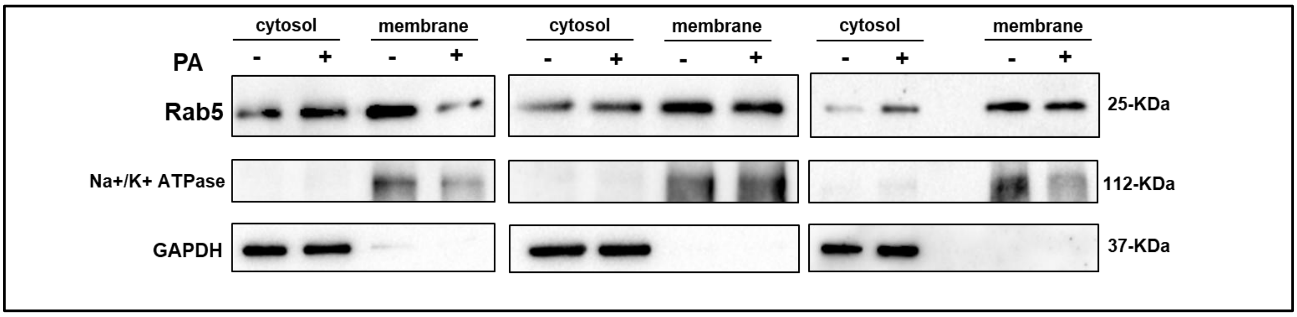

Supplemental Figure 14. Western blot analysis of Rab5 in cytosol and membrane-enriched fraction. An anti-GAPDH antibody was used to control the purity of membrane-enriched fractions and the protein loading of cytosol, and an anti-Na+/K+ATPase antibody to control the purity of cytosol and the protein loading of membrane-enriched fractions. All samples are shown.

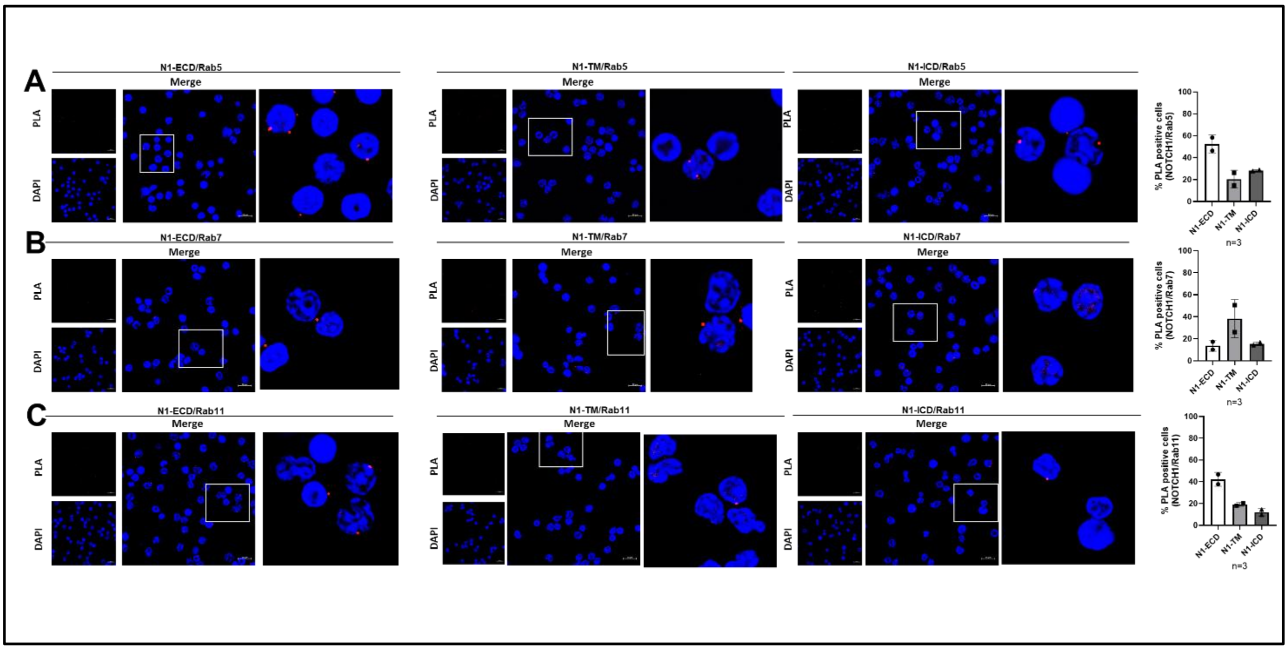

Supplemental Figure 15. Analysis of NOTCH1 colocalization with Rab5, Rab7 and Rab11 markers in *NOTCH1-* mutated CLL cells. PLA was performed in *NOTCH1-* mutated CLL cells (n=2) to detect the interaction of N1-ECD, N1-TM and N1-ICD with Rab5 (A), Rab7 (B) and Rab11 (C). Left panels, confocal microscopy images from one representative CLL sample. PLA signals indicating interactions are visualized by red spots and nuclei by DAPI staining. Images were acquired using confocal microscopy with a 63x oil immersion and 1.4 NA objective. Scale bar 10μM. Right panels, bar graphs with individual data points show mean ± SEM of the percentage of PLA positive cells in two samples. Each dot represents one patient sample. For each patient, five fields per condition were analyzed and averaged to obtain a single value.

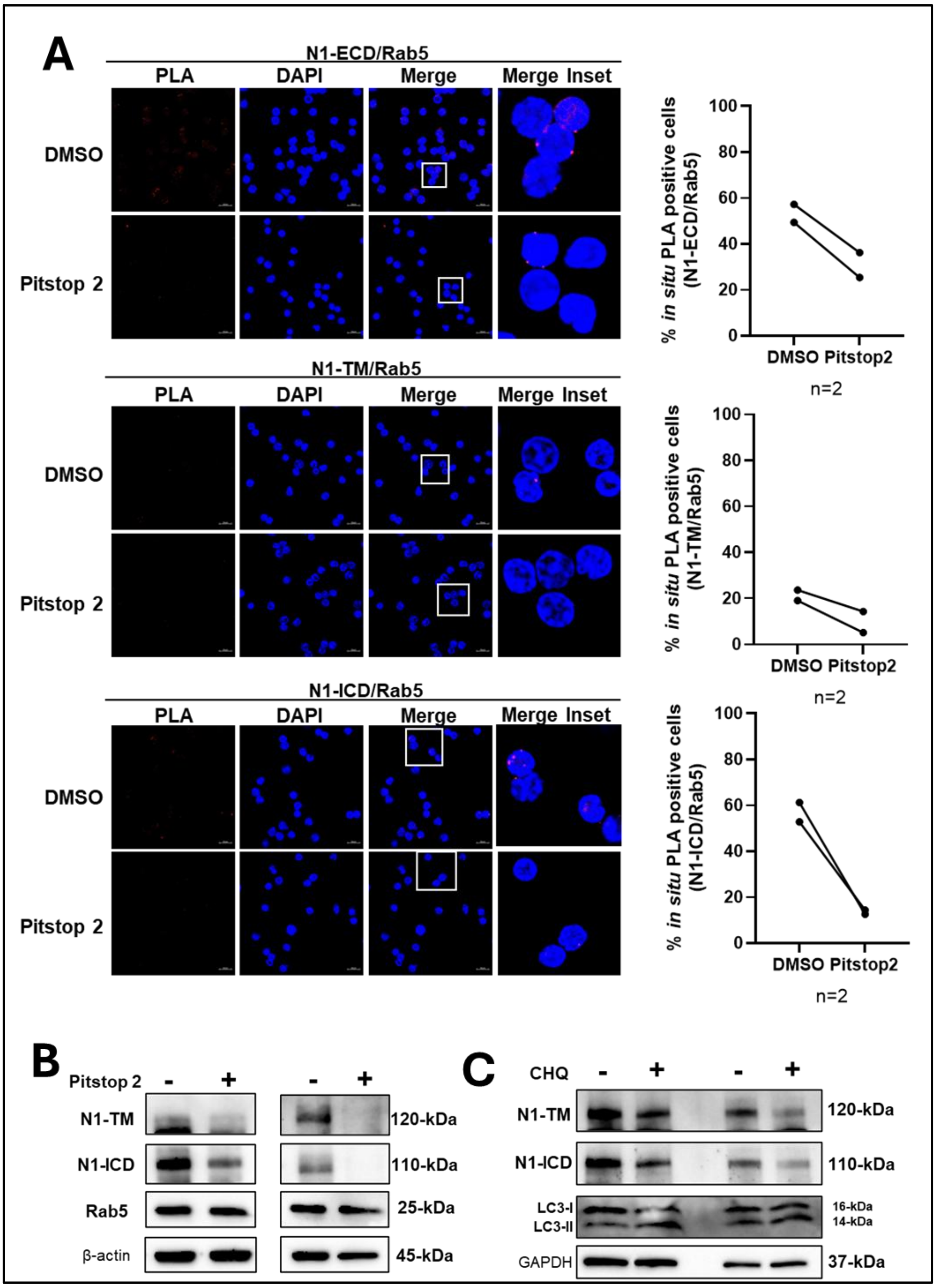

Supplemental Figure 16. Targeting endocytosis and endosomal acidification reduces N1-ICD levels in *NOTCH1*-mutated CLL cells. (A–B) CLL cells from *NOTCH1*-mutated CLL cells (n=2) were treated with the endocytosis inhibitor Pitstop-2 or DMSO as control for 30 minutes. (A) PLA performed to detect the interactions of N1-ECD, N1-TM and N1-ICD with the early endosome marker Rab5. Left panels, confocal microscopy images from one representative sample. PLA signals indicating interactions are visualized by red spots and nuclei by DAPI staining. Images were acquired using confocal microscopy with a 63× oil immersion objective (NA 1.4). Scale bar: 10 µm. Right panels, paired dot plots showing the percentage of PLA-positive cells from two samples. Each dot represents one patient sample

and lines connect paired conditions (DMSO vs Pitstop-2). For each patient, five fields per condition were analyzed and averaged to obtain a single value. (B) Western blot analysis of N1-TM, N1-ICD and RAB5 expression. β-actin was used as loading control. Two CLL samples are shown. (C) CLL cells were cultured for 5 hours with 50µM the endosomal acidification inhibitor chloroquine (CHQ) or complete medium as control before Western blot analysis of N1-TM and N1-ICD and of LC3-II accumulation. GAPDH was used as loading control. Two CLL samples are shown.

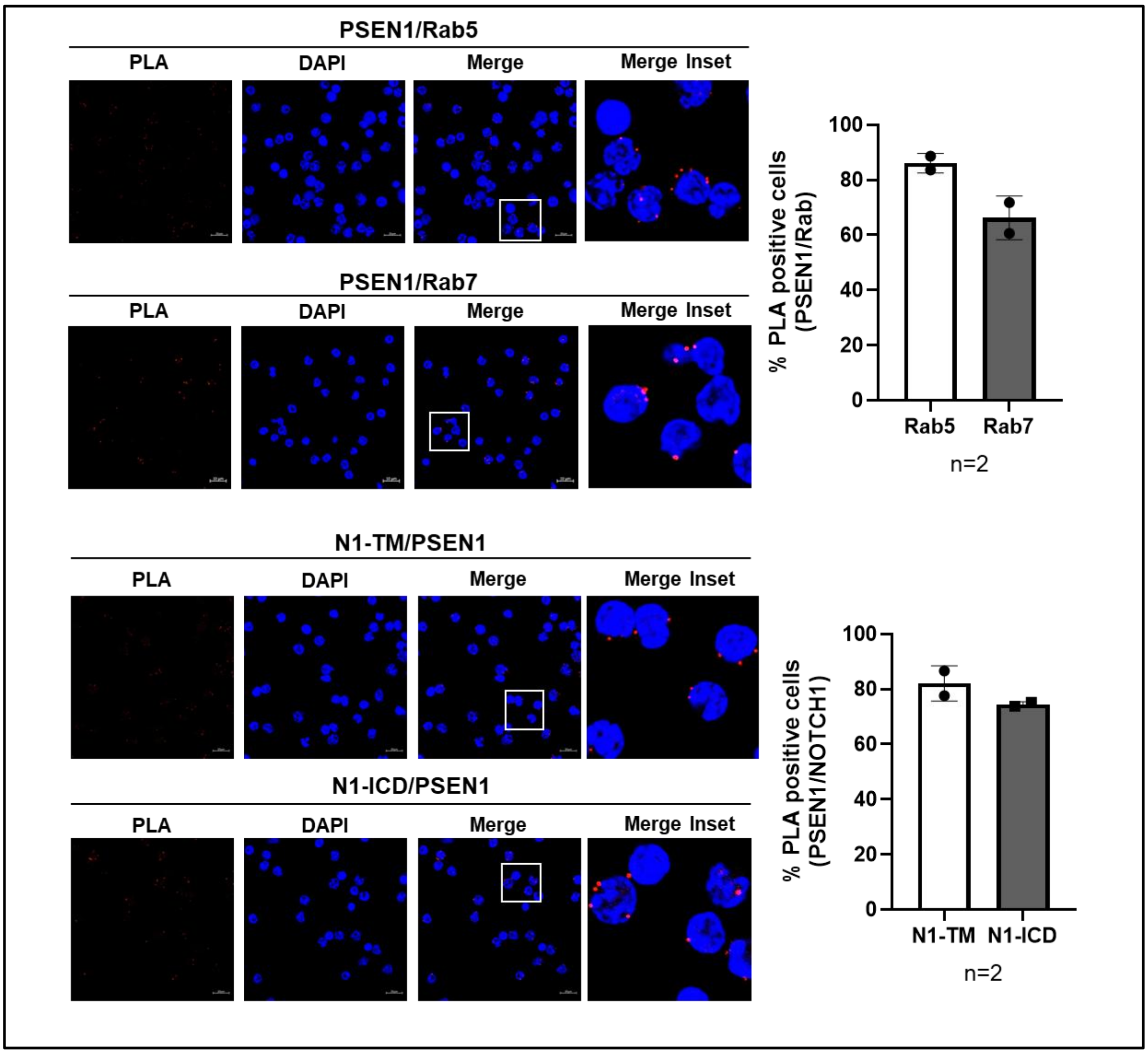

Supplemental Figure 17. Colocalization of PSEN1 with Rab5 and Rab7 and with N1-TM and N1-ICD in *NOTCH1*-mutated CLL cells. PLA was performed in *NOTCH1*-mutated CLL cells (n=2) to detect the interactions of PSEN1 with Rab5 and Rab7 (upper panels) and with N1-TM and N1-ICD (bottom panel). Confocal microscopy images from one representative sample. PLA signals indicating interactions are visualized by red spots and nuclei by DAPI staining. Images were acquired using confocal microscopy with a 63x oil immersion and 1.4 NA objective. Scale bar 10 µm. Right, bar graphs showing mean±SEM of the percentage of PLA positive cells in two CLL samples. Each dot represents one patient sample. For each patient, five fields per condition were analyzed and averaged to obtain a single value.

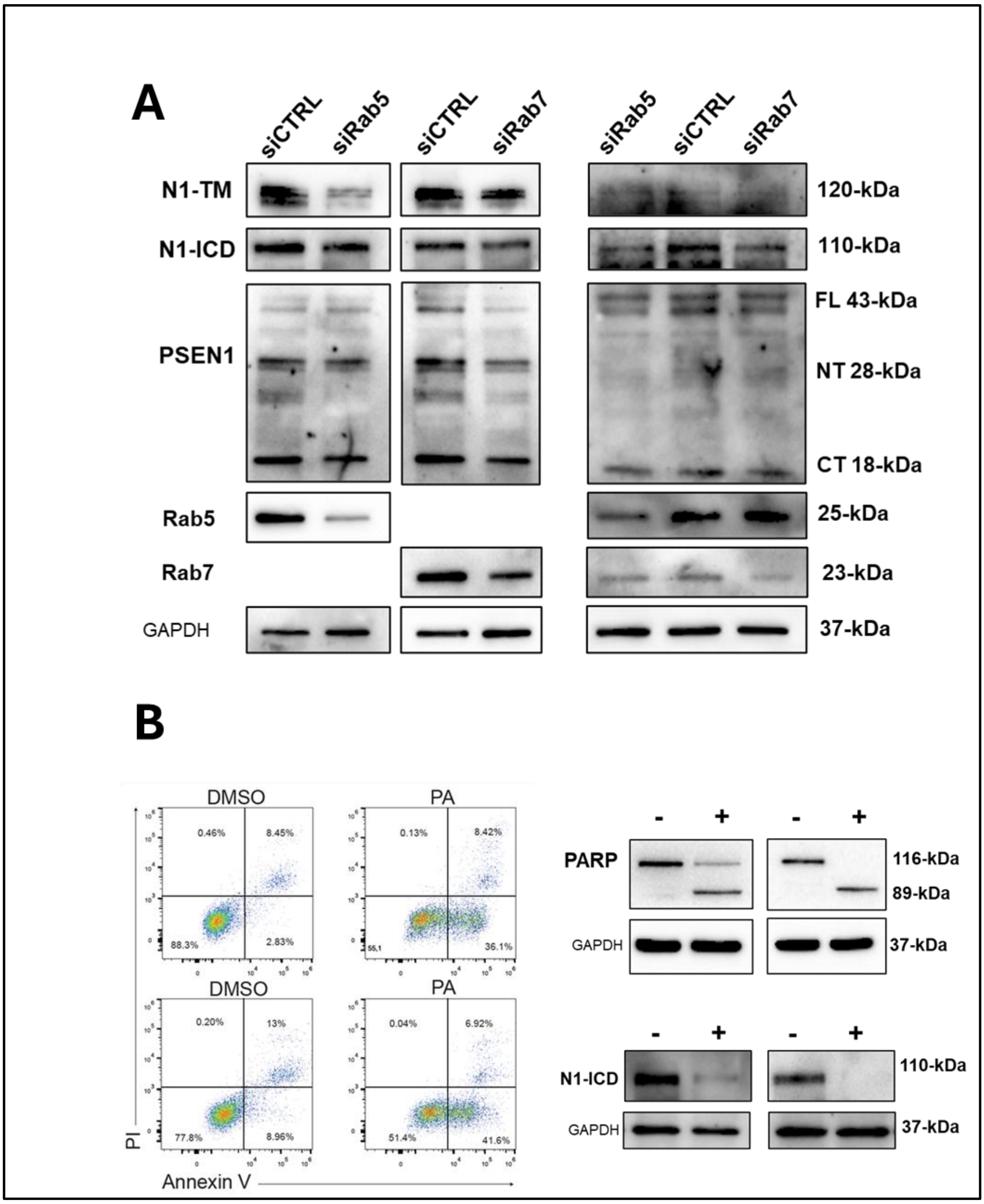

Supplemental Figure 18 Rab targeting strategies reduce N1-ICD levels in *NOTCH1*-mutated CLL cells. (A) Western blot analysis of N1-TM, N1-ICD and PSEN1 in *NOTCH1*-mutated CLL cells (n=2) transfected with control siRNA, Rab5 siRNA (siRab5) or Rab7 siRNA (siRab7). Silencing efficiency was assessed by analyzing Rab5 or Rab7 expression. Analysis of PSEN1 shows the full-length (FL) and the N-terminal (NT) and the C-terminal (CT) fragments derived from PSEN1 proteolytic activation. Protein loading was assessed by using an anti-GAPDH antibody. (B) Cell viability/apoptosis and N1-ICD expression in two *NOTCH1*-mutated samples treated with psoromic acid (PA) as described in Figure 6. Left panel, results of cell viability and apoptosis analysis evaluated by of Annexin V/PI staining and flow cytometry. Plots from both patients are shown. Right panel, western blot analysis of PARP cleavage and N1-ICD expression. GAPDH was analyzed as loading control. Blots from both patients are shown.

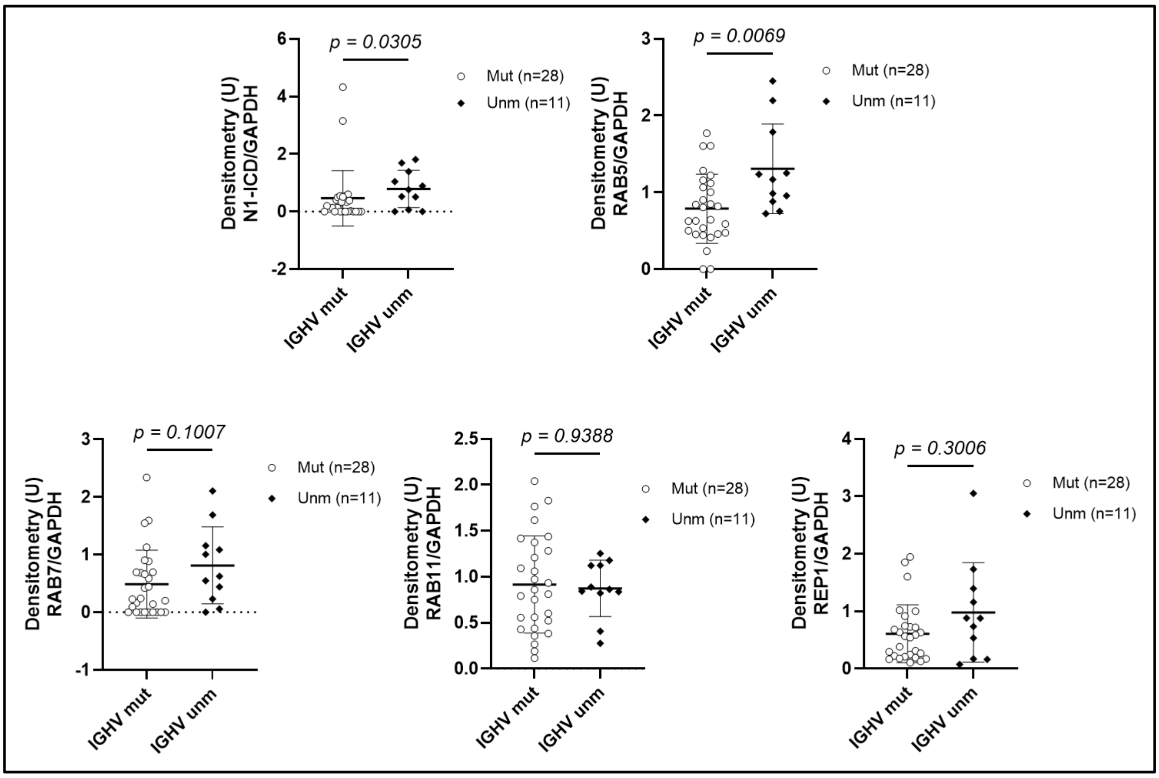

Supplemental Figure 19. Association of N1-ICD, Rab proteins and REP1 with *IGHV* mutational status in CLL. Dot plots showing the distribution of N1-ICD, RAB5, RAB7, RAB11and REP1 expression levels in CLL samples stratified according to *IGHV* mutational status (*IGHV*-unmutated vs *IGHV*-mutated). Each dot represents an individual patient value calculated as a protein to GAPDH ratio and expressed as densitometric units (U). Statistical analysis was performed using the Mann–Whitney test; *p* value is indicated above each graph.

Supplemental Methods

Next-generation sequencing (NGS)

Targeted NGS was performed using a Custom Illumina enrichment panel and sequencing on the MiSeq platform (RUO). Full coding regions of *ATM, NOTCH1, MYD88, TP53, BTK, BCL2, PLCG2, and BCOR* were analyzed, together with hotspot regions in *BIRC3* (exons 6–8), *SF3B1* (exons 13–18), *FBXW7* (exons 8–12), *KLHL6* (exon 1), *POT1* (exons 6–9), *XPO1* (exons 15–16), *RPS15* (exon 4), *MED12* (exons 1–2), and *EGR2* (exon 2). NGS libraries were prepared according to the user guide from 300 ng of genomic DNA and their quality was assessed using the Quality Control Agilent kit (Tape Station, Agilent, Santa Clara, CA; USA and Qubit, Thermo Fisher Scientific, Eugene, OR, USA). Sequencing was performed using Illumina MiSeq. Variants with allele frequency ≥5% were considered. Mean coverage depth was >1000×, with >95% target coverage. Data were analyzed using NextGENe software.

CLL co-culture with OP9-DLL1 stromal cells

Primary CLL cells were co-cultured with the murine stromal cell line OP9-DLL1, which constitutively expresses the NOTCH ligand DLL1, to induce NOTCH1 activation. OP9-DLL1 cells were maintained in α-MEM supplemented with 20% fetal bovine serum (FBS) and antibiotics. For co-culture experiments, OP9-DLL1 cells were seeded in 6-well plates and allowed to reach confluence, after which primary CLL cells were added at a density of 2×10^6 cells per well. After a 6-hour incubation, non-adherent CLL cells were carefully collected and processed for downstream analyses

Flow cytometry

NOTCH1 surface expression was assessed using an anti-human NOTCH1 antibody (APC or PE conjugated; Supplemental Table 2) or the corresponding isotype control. Data were analyzed by gating on live singlet $CD19^+/CD5^+$ cells and are reported as the percentage of positive cells. Surface levels of transferrin and transferrin receptor (CD71) following Pitstop-2 treatment were evaluated using Alexa Fluor 488–conjugated transferrin (Thermo Fisher Scientific) and an anti-human CD71-APC-AF700 antibody (Supplemental Table 2), respectively. Data were analyzed on live singlet $CD19^+/CD5^+$ cells, and median

fluorescence intensity (MFI) values were compared between Pitstop-2– and DMSO-treated samples.
